# Supplementary material for: Variations in antibiotic prescribing among village doctors in a rural region of Shandong province, China: a cross-sectional analysis of prescriptions
Source: BMJ Open. 2020 Jun 1;10(6):e036703. doi: 10.1136/bmjopen-2019-036703 (PMC7265041; doi:10.1136/bmjopen-2019-036703)
Supplement: Supplementary data [file bmjopen-2019-036703supp002.pdf]

Supplementary material: STROBE Statement—checklist of items that should be included in reports of observational studies, for: Variations in antibiotic prescribing among village doctors in a rural region of Shandong province, China: a cross-sectional analysis of prescriptions

Oliver J. Dyar, Yang Ding, Jia Yin, Sun Qiang[+], Cecilia Stålsby Lundborg

[+]Corresponding author: School of Health Care Management, Shandong University, Jinan, Shandong, China. Tel: +86-531-88382376; Fax: +86-531-88382693; E-mail: qiangs@sdu.edu.cn

	Item No.	Recommendation	Page No.	Relevant text from manuscript
**Title and abstract**	1	(*a*) Indicate the study's design with a commonly used term in the title or the abstract	1	"Variations in antibiotic prescribing among village doctors in a rural region of Shandong province, China: a cross-sectional analysis of prescriptions"
		(*b*) Provide in the abstract an informative and balanced summary of what was done and what was found	2	As per abstract
**Introduction**				
Background/rationale	2	Explain the scientific background and rationale for the investigation being reported	3	e.g. "These studies have not been capable of investigating the extent of variability in practices that exist"
Objectives	3	State specific objectives, including any prespecified hypotheses	3	e.g. "Our aims were to assess if there was overall evidence of irrational antibiotic use, and whether there was significant variability between the practices of individual prescribers in this small, homogenous region."

Dyar OJ, *et al. BMJ Open* 2020; 10:e036703. doi: 10.1136/bmjopen-2019-036703

**Methods**				
Study design	4	Present key elements of study design early in the paper	3	e.g. "We conducted a prospective observational analysis of outpatient prescriptions from January 2015 to July 2017 at eight village clinics clustered around a single town in Z County, Shandong province."
Setting	5	Describe the setting, locations, and relevant dates, including periods of recruitment, exposure, follow-up, and data collection	3-4	e.g. sections on study design and setting, data collection
Participants	6	(*a*) *Cohort study*—Give the eligibility criteria, and the sources and methods of selection of participants. Describe methods of follow-up<br>*Case-control study*—Give the eligibility criteria, and the sources and methods of case ascertainment and control selection. Give the rationale for the choice of cases and controls<br>*Cross-sectional study*—Give the eligibility criteria, and the sources and methods of selection of participants	3-4	e.g. description of how village clinics were selected as part of broader IMPACT research programme
		(*b*) *Cohort study*—For matched studies, give matching criteria and number of exposed and unexposed<br>*Case-control study*—For matched studies, give matching criteria and the number of controls per case	Not relevant	
Variables	7	Clearly define all outcomes, exposures, predictors, potential confounders, and effect modifiers. Give diagnostic criteria, if applicable	4-5	e.g. descriptions of coding of prescriptions; definitions of antibiotic prescribing rate, multiple antibiotics prescribing rate, parenteral antibiotic prescribing rate; European quality indicators comparison
Data sources/ measurement	8*	For each variable of interest, give sources of data and details of methods of assessment (measurement). Describe comparability of assessment methods if there is more than one group	4	e.g. descriptions of sampling of prescriptions under "data collection" and also data

Dyar OJ, *et al. BMJ Open* 2020; 10:e036703. doi: 10.1136/bmjopen-2019-036703

				management
Bias	9	Describe any efforts to address potential sources of bias	4	e.g. "Prescriber-level analyses were limited to the 20/23 doctors with ≥50 AURI prescriptions during the study period, to ensure that the antibiotic prescribing rates calculated for each individual doctor were sufficiently representative of their practice."
Study size	10	Explain how the study size was arrived at	4	e.g. descriptions of how village clinics were selected as part of broader IMPACT research programme

Quantitative variables	11	Explain how quantitative variables were handled in the analyses. If applicable, describe which groupings were chosen and why	4	e.g. "A category of *likely viral acute upper respiratory tract infections* (AURI) was created by grouping diagnoses of J00 (acute nasopharyngitis [common cold]) and J06.9 (acute upper respiratory infection, unspecified)."
Statistical methods	12	(*a*) Describe all statistical methods, including those used to control for confounding	4-5	e.g. section on data management and analyses
		(*b*) Describe any methods used to examine subgroups and interactions	4-5	e.g. "Comparisons were also made against antibiotic prescribing quality indicators developed in Europe[14] for a subset of clinical diagnoses and patient ages."
		(*c*) Explain how missing data were addressed	4	e.g. "Prescriptions from one doctor who did not complete the

Dyar OJ, *et al. BMJ Open* 2020; 10:e036703. doi: 10.1136/bmjopen-2019-036703

				questionnaire were removed (55 in total)."
		(*d*) *Cohort study*—If applicable, explain how loss to follow-up was addressed<br>*Case-control study*—If applicable, explain how matching of cases and controls was addressed<br>*Cross-sectional study*—If applicable, describe analytical methods taking account of sampling strategy	Not relevant	
		(*e*) Describe any sensitivity analyses	Not conducted	
**Results**				
Participants	13*	(a) Report numbers of individuals at each stage of study—eg numbers potentially eligible, examined for eligibility, confirmed eligible, included in the study, completing follow-up, and analysed	5	e.g. "In total 14471 prescriptions from 23 prescribers from January 2015 to July 2017 were included in the analyses. This included an average of 58 prescriptions per month per village clinic (mean range from 47 to 62).". Additional text in methods and in discussion is relevant.
		(b) Give reasons for non-participation at each stage	4	e.g. "Of the three doctors with fewer than 50 AURI prescriptions, two retired from clinical practice near the beginning of the study period (616 and 720), and one newly started working at the specific village clinic at the end of the study period (618)."
		(c) Consider use of a flow diagram	Not sufficiently valuable	
Descriptive data	14*	(a) Give characteristics of study participants (eg demographic, clinical, social) and information on exposures and potential confounders	7-8	E.g. section on "prescriber demographics" and Table 3

		(b) Indicate number of participants with missing data for each variable of interest	Not relevant	
		(c) *Cohort study*—Summarise follow-up time (eg, average and total amount)	Not relevant	
Outcome data	15*	*Cohort study*—Report numbers of outcome events or summary measures over time		
		Case-control study—Report numbers in each exposure category, or summary measures of exposure		
		Cross-sectional study—Report numbers of outcome events or summary measures	5-6	e.g. "Overall, 40.3% of the prescriptions (5833/14471) contained at least one antibiotic." Also, table 1.
Main results	16	(*a*) Give unadjusted estimates and, if applicable, confounder-adjusted estimates and their precision (eg, 95% confidence interval). Make clear which confounders were adjusted for and why they were included	5-9	e.g. "Overall, respiratory tract infections accounted for 68.4% (3991/5833) and gastrointestinal conditions for 14.0% (815/5833) of all prescriptions containing at least one antibiotic.", and several other results. Due to the small number of prescribers, ranges in prescribing rates are provided, for example in section on "Variations at the prescriber-level" and Figure 2.
		(*b*) Report category boundaries when continuous variables were categorized	Not relevant	
		(*c*) If relevant, consider translating estimates of relative risk into absolute risk for a meaningful time period	Not relevant	
Other analyses	17	Report other analyses done—eg analyses of subgroups and interactions, and sensitivity analyses	7, 9	e.g. "Comparison with European quality indicators" and "Comparisons between high and low antibiotic prescribers"
**Discussion**				
Key results	18	Summarise key results with reference to study objectives	10-11	e.g. "We prospectively investigated patterns of antibiotic prescriptions over a two-and-a-half-year period in

				village clinics in rural Shandong province in order to assess evidence of irrational antibiotic use and variability in prescribing practices at the level of the individual prescriber."
Limitations	19	Discuss limitations of the study, taking into account sources of potential bias or imprecision. Discuss both direction and magnitude of any potential bias	12-13	e.g. "A common limitation of studies using prescription data is the inability to verify diagnoses. To restrict the impact of this limitation we restricted the individual-level analyses to AURI diagnoses, but we cannot guarantee that misclassifications did not occur, particularly given there is currently no incentive for doctors to correctly classify diagnoses." and entire section on methodological considerations
Interpretation	20	Give a cautious overall interpretation of results considering objectives, limitations, multiplicity of analyses, results from similar studies, and other relevant evidence	13	e.g. "Together our results provide evidence that even in a small homogenous setting in rural China, variations in individual prescriber practices are significant, and they need to be accounted for in the development of targets and interventions to improve antibiotic use."
Generalisability	21	Discuss the generalisability (external validity) of the study results	13	e.g. "We suspect this finding is likely to be generalizable to other areas in rural Shandong province as

				a minimum, but also to other areas in eastern rural China, where the healthcare system is very similar in terms of structure, regulations and staffing.”
**Other information**				
Funding	22	Give the source of funding and the role of the funders for the present study and, if applicable, for the original study on which the present article is based	13	“This work was supported by the Swedish Research Council (grant number D0879801) and National Natural Science Foundation of China (grant number 81361138021).”

*Give information separately for cases and controls in case-control studies and, if applicable, for exposed and unexposed groups in cohort and cross-sectional studies.

Note: An Explanation and Elaboration article discusses each checklist item and gives methodological background and published examples of transparent reporting. The STROBE checklist is best used in conjunction with this article (freely available on the Web sites of PLoS Medicine at http://www.plosmedicine.org/, Annals of Internal Medicine at http://www.annals.org/, and Epidemiology at http://www.epidem.com/). Information on the STROBE Initiative is available at www.strobe-statement.org.

Dyar OJ, *et al. BMJ Open* 2020; 10:e036703. doi: 10.1136/bmjopen-2019-036703
