# Supplementary material for: Variations in antibiotic prescribing among village doctors in a rural region of Shandong province, China: a cross-sectional analysis of prescriptions
Source: BMJ Open. 2020 Jun 1;10(6):e036703. doi: 10.1136/bmjopen-2019-036703 (PMC7265041; doi:10.1136/bmjopen-2019-036703)
Supplement: Supplementary data [file bmjopen-2019-036703supp001.pdf]

Supplementary tables for:

Variations in antibiotic prescribing among village doctors in a rural region of Shandong province, China: a cross-sectional analysis of prescriptions

Oliver J. Dyar, Yang Ding, Jia Yin, Sun Qiang+, Cecilia Stålsby Lundborg

+Corresponding author: School of Health Care Management, Shandong University, Jinan, Shandong, China. Tel: +86-531-88382376; Fax: +86-531-88382693; E-mail: qiangs@sdu.edu.cn

Appendix table 1. Class and agent of antibiotic prescribing for AURI (N = 3818)

Antibiotic class	Frequency	%	Antibiotic agent	Frequency	%
J01AA Tetracyclines	3	0.1	J01AA02 Doxycycline	3	0.1
J01CA Penicillins with Extended Spectrum	***1025***	***26.8***	J01CA01 Ampicillin	8	0.2
			J01CA04 Amoxicillin	***1017***	***26.6***
J01CE Beta-Lactamase Sensitive Penicillins	49	1.3	J01CE01 Benzylpenicillin	35	0.9
			J01CE02 Phenoxymethylpenicillin	14	0.4
J01CR Combinations of Penicillins, Incl. Beta-Lactamase Inhibitors	79	2.1	J01CR02 Amoxicillin and enzyme inhibitor	79	2.1
J01DB First-Generation Cephalosporins	***603***	***15.8***	***J01DB01 Cefalexin***	***323***	***8.5***
			J01DB09 Cefradine	280	7.3
J01DC Second-Generation Cephalosporins	***272***	***7.1***	J01DC02 Cefuroxime	272	7.1
J01DD Third-Generation Cephalosporins	255	6.7	J01DD01 Cefotaxime	193	5.1
			J01DD08 Cefixime	62	1.6
J01EE Combinations of Sulfonamides and Trimethoprim, Incl. Derivatives	1	0.0	J01EE01 Sulfamethoxazole	1	0.0
J01FA Macrolides	***516***	***13.5***	***J01FA06 Roxithromycin***	***317***	***8.3***
			J01FA09 Clarithromycin	7	0.2
			J01FA10 Azithromycin	192	5.0
J01FF Lincosamides	343	9.0	J01FF01 Clindamycin	45	1.2
			J01FF02 Lincomycin	***298***	***7.8***
J01GB Other Aminoglycosides	166	4.3	J01GB03 Gentamicin	37	1.0
			J01GB06 Amikacin	129	3.4
J01MA Fluoroquinolones	***423***	***11.1***	J01MA02 Ciprofloxacin	64	1.7
			J01MA06 Norfloxacin	25	0.7
			J01MA12 Levofloxacin	***334***	***8.7***
J01MB Other Quinolones	20	0.5	J01MB04 Pipemidic Acid	20	0.5
J01XD Imidazole Derivatives	58	1.5	J01XD01 Metronidazole	8	0.2
			J01XD02 Tinidazole	50	1.3
J01XE Nitrofuran Derivatives	3	0.1	J01XE01 Nitrofurantoin	3	0.1
J01XX Other Antibacterials	2	0.1	J01XX01 Fosfomycin	2	0.1

Appendix Table 2. Additional antibiotic prescribing rate indicators for individual prescribers

Prescriber	Clinic	Sex	Age	% of respiratory tract infection diagnoses coded as AURI			APR of AURI in 2015			APR of AURI in 2016			APR of potentially bacterial URTIs			APR of gastroenteritis, diarrhoea and colitis		
				n	N	%	n	N	%	n	N	%	n	N	%	n	N	%
101	BFZG	2	46	377	647	58.3	151	181	83.4	70	142	49.3	56	61	91.8	58	62	93.5
102	BFZG	1	52	56	76	73.7	NA	NA	NA	NA	NA	NA	9	9	100.0	NA	NA	NA
203	CS	1	59	167	251	66.5	12	26	46.2	40	71	56.3	63	83	75.9	2	2	100.0
204	CS	1	46	73	126	57.9	17	22	77.3	21	28	75.0	50	52	96.2	10	10	100.0
205	CS	2	50	111	141	78.7	37	63	58.7	19	24	79.2	20	27	74.1	3	3	100.0
206	CS	1	63	173	216	80.1	57	87	65.5	73	86	84.9	32	39	82.1	10	10	100.0
307	DS	2	40	520	605	86.0	88	199	44.2	38	160	23.8	39	51	76.5	64	111	57.7
308	DS	1	67	217	251	86.5	57	132	43.2	29	84	34.5	20	28	71.4	33	70	47.1
409	FJZZ	1	63	134	136	98.5	54	77	70.1	45	57	78.9	NA	NA	NA	21	46	45.7
410	FJZZ	1	40	199	200	99.5	82	123	66.7	64	76	84.2	NA	NA	NA	25	48	52.1
411	FJZZ	1	43	124	146	84.9	NA	NA	NA	26	38	68.4	7	9	77.8	12	15	80.0
412	FJZZ	1	60	271	285	95.1	98	108	90.7	92	118	78.0	2	2	100.0	38	69	55.1
513	QD	1	46	117	124	94.4	76	89	85.4	27	28	96.4	NA	NA	NA	4	4	100.0
514	QD	1	52	578	597	96.8	113	123	91.9	168	222	75.7	2	2	100.0	31	35	88.6
515	QD	1	65	181	192	94.3	80	102	78.4	55	79	69.6	NA	NA	NA	16	20	80.0
616	XJG	1	63	19	33	57.6	4	14	28.6	1	5	20.0	6	9	66.7	2	3	66.7
617	XJG	1	39	415	546	76.0	76	117	65.0	98	163	60.1	22	31	71.0	69	100	69.0
618	XJG	1	37	12	13	92.3	NA	NA	NA	8	12	66.7	0	1	0.0	12	17	70.6
719	YJ	1	42	386	437	88.3	104	153	68.0	78	129	60.5	1	1	100.0	33	38	86.8
720	YJ	1	63	3	3	100.0	0	1	0.0	1	2	50.0	NA	NA	NA	NA	NA	NA
721	YJ	1	36	408	416	98.1	114	152	75.0	108	163	66.3	1	1	100.0	30	35	85.7
822	YJZZ	1	68	378	407	92.9	127	184	69.0	99	194	51.0	9	20	45.0	46	50	92.0
823	YJZZ	2	55	258	335	77.0	69	95	72.6	30	67	44.8	23	35	65.7	38	38	100.0

Footnote: APR = antibiotic prescribing rate; AURI = likely viral acute upper respiratory tract infections; potentially bacterial upper respiratory tract infections (URTIs) = prescription containing and diagnosis of pharyngitis, tonsillitis or laryngopharyngitis. Rows in grey are not included in the individual prescriber-level analyses in the main text due to contributing <50 AURI prescriptions (prescribers 616, 618, 720) and due to an error in the coding of prescriber (prescribers 101, 102). 1 = male; 2 = female.

Appendix table 3. Most common antibiotic agents prescribed for AURI at individual prescriber level

Presc riber	Clinic	Sex	Age	1st most common antibiotic prescribed for AURI, name (no.)			2nd most common antibiotic prescribed for AURI, name (no.)			3rd most common antibiotic prescribed for AURI, name (no.)			Sum top 3	Total no. abx	% abx that are top three	No. abx agents total
101	BFZG	2	46	J01CA04	Amoxicillin	126	J01DB01	Cefalexin	35	J01FA06	Roxithromycin	31	192	247	77.7	13
102	BFZG	1	52	J01CA04	Amoxicillin	9							9	9	100.0	1
203	CS	1	59	J01MA12	Levofloxacin	25	J01DB09	Cefradine	21	J01CA04	Amoxicillin	16	62	93	66.7	13
204	CS	1	46	J01MA12	Levofloxacin	16	J01CA04	Amoxicillin	13	J01DD01	Cefotaxime	12	41	56	73.2	11
205	CS	2	50	J01CA04	Amoxicillin	21	J01DB09	Cefradine	12	J01MA12	Levofloxacin	12	45	77	58.4	12
206	CS	1	63	J01CA04	Amoxicillin	46	J01DB09	Cefradine	23	J01DB01	Cefalexin	19	88	130	67.7	11
307	DS	2	40	J01CA04	Amoxicillin	35	J01MA02	Ciprofloxacin	23	J01FA06	Roxithromycin	20	78	172	45.3	20
308	DS	1	67	J01CA04	Amoxicillin	24	J01DB01	Cefalexin	15	J01DB09<br>J01DC02<br>J01MA02	Cefradine<br>Cefuroxime<br>Ciprofloxacin	9<br>9<br>9	48	86	55.8	17
409	FJZZ	1	63	J01CA04	Amoxicillin	20	J01DB09	Cefradine	17	J01FA06	Roxithromycin	16	53	99	53.5	17
410	FJZZ	1	40	J01CA04	Amoxicillin	25	J01FA06	Roxithromycin	23	J01FF02	Lincomycin	23	71	146	48.6	18
411	FJZZ	1	43	J01FF02	Lincomycin	22	J01CA04	Amoxicillin	18	J01MA12	Levofloxacin	12	52	85	61.2	11
412	FJZZ	1	60	J01CA04	Amoxicillin	60	J01DB09	Cefradine	46	J01FA06	Roxithromycin	39	145	231	62.8	16
513	QD	1	46	J01GB06	Amikacin	33	J01DD01	Cefotaxime	28	J01MA12	Levofloxacin	28	89	103	86.4	16
514	QD	1	52	J01CA04	Amoxicillin	140	J01FF02	Lincomycin	137	J01DB09	Cefradine	28	305	407	74.9	21
515	QD	1	65	J01CA04	Amoxicillin	53	J01FF02	Lincomycin	27	J01GB06	Amikacin	15	95	135	70.4	18
616	XJG	1	63	J01CA04	Amoxicillin	1	J01CR02	Amoxicillin and enzyme inhibitor	1	J01DB01	Cefalexin	1	3	5	60.0	6
617	XJG	1	39	J01FA10	Azithromycin	49	J01CA04	Amoxicillin	46	J01FA06	Roxithromycin	42	137	265	51.7	19
618	XJG	1	37	J01CA04	Amoxicillin	2	J01DC02	Cefuroxime	2	J01DB09	Cefradine	1	5	8	62.5	7
719	YJ	1	42	J01CA04	Amoxicillin	86	J01DC02	Cefuroxime	43	J01DB01	Cefalexin	39	168	249	67.5	20
720	YJ	1	63	J01GB06	Amikacin	1	J01AA02						1	1	100.0	1
721	YJ	1	36	J01CA04	Amoxicillin	68	J01MA12	Levofloxacin	59	J01FA06	Roxithromycin	43	170	276	61.6	20
822	YJZZ	1	68	J01CA04	Amoxicillin	112	J01FA06	Roxithromycin	26	J01DC02	Cefuroxime	25	163	226	72.1	17

823	YJZZ	2	55	J01CA04	Amoxicillin	75	J01DB09	Cefradine	18	J01DB01	Cefalexin	14	107	131	81.7	13

Footnote: AURI = likely viral acute upper respiratory tract infections; abx = antibiotics; 1 = male; 2 = female. Rows in grey are not included in the individual prescriber-level analyses in the main text due to contributing <50 AURI prescriptions (prescribers 616, 618, 720) and due to an error in the coding of prescriber (prescribers 101, 102).

Appendix table 4. Antibiotic use for AURI at level of individual prescriber

Prescriber	Clinic	Sex	Age	Patient age			Female patients		APR for ALL prescriptions			APR for AURI			MPR for AURI			PAPR for AURI			AURI with analgesic or anti-inflammatory		
				Mean	Min	Max	n	%	n	N	%	n	N	%	n	N	%	n	N	%	n	N	%
101	BFZG	2	46	48	2	90	152	40.3	794	1674	47.4	247	377	65.5	24	247	9.7	10	247	4.0	260	377	69.0
102	BFZG	1	52	50	13	78	19	33.9	37	185	20.0	9	56	16.1	0	9	0.0	0	9	0.0	35	56	62.5
203	CS	1	59	53	5	81	53	31.7	226	598	37.8	93	167	55.7	3	93	3.2	3	93	3.2	127	167	76.0
204	CS	1	46	56	36	75	37	50.7	165	283	58.3	56	73	76.7	12	56	21.4	25	56	44.6	34	73	46.6
205	CS	2	50	52	9	80	50	45.0	142	357	39.8	77	111	69.4	1	77	1.3	5	77	6.5	66	111	59.5
206	CS	1	63	49	5	86	62	35.8	246	560	43.9	130	173	75.1	2	130	1.5	13	130	10.0	109	173	63.0
307	DS	2	40	50	1	90	206	39.6	372	1255	29.6	172	520	33.1	14	172	8.1	25	172	14.5	229	520	44.0
308	DS	1	67	47	4	89	95	43.8	171	576	29.7	86	217	39.6	7	86	8.1	17	86	19.8	78	217	35.9
409	FJZZ	1	63	54	5	84	64	47.8	138	328	42.1	99	134	73.9	12	99	12.1	28	99	28.3	55	134	41.0
410	FJZZ	1	40	53	3	86	95	47.7	191	405	47.2	146	199	73.4	39	146	26.7	57	146	39.0	80	199	40.2
411	FJZZ	1	43	56	10	83	57	46.0	144	365	39.5	85	124	68.5	11	85	12.9	30	85	35.3	61	124	49.2
412	FJZZ	1	60	54	5	90	137	50.6	329	654	50.3	231	271	85.2	24	231	10.4	41	231	17.7	134	271	49.4
513	QD	1	46	47	10	85	57	48.7	138	197	70.1	103	117	88.0	62	103	60.2	64	103	62.1	20	117	17.1
514	QD	1	52	49	2	92	286	49.5	528	1111	47.5	407	578	70.4	47	407	11.5	179	407	44.0	205	578	35.5
515	QD	1	65	49	3	83	86	47.5	196	417	47.0	135	181	74.6	27	135	20.0	51	135	37.8	83	181	45.9
616	XJG	1	63	56	5	82	4	21.1	34	101	33.7	5	19	26.3	1	5	20.0	2	5	40.0	7	19	36.8
617	XJG	1	39	44	1	87	156	37.6	536	1634	32.8	265	415	63.9	24	265	9.1	71	265	26.8	173	415	41.7
618	XJG	1	37	44	10	63	4	33.3	27	91	29.7	8	12	66.7	1	8	12.5	1	8	12.5	6	12	50.0
719	YJ	1	42	54	2	90	178	46.1	416	923	45.1	249	386	64.5	61	249	24.5	47	249	18.9	225	386	58.3
720	YJ	1	63	60	34	73	3	100.0	1	5	20.0	1	3	33.3	0	1	0.0	1	1	100.0	2	3	66.7
721	YJ	1	36	57	4	91	150	36.8	415	928	44.7	276	408	67.6	77	276	27.9	70	276	25.4	202	408	49.5
822	YJZZ	1	68	50	2	87	171	45.2	338	1038	32.6	226	378	59.8	35	226	15.5	38	226	16.8	159	378	42.1
823	YJZZ	2	55	48	2	86	119	46.1	249	786	31.7	131	258	50.8	7	131	5.3	7	131	5.3	141	258	54.7

Footnote: APR = antibiotic prescribing rate; MPR = multiple antibiotic prescribing rate; PAPR = parenteral antibiotic prescribing rate; AURI = likely viral acute upper respiratory tract infections; 1 = male; 2 = female. Rows in grey are not included in the individual prescriber-level analyses in the main text due to contributing <50 AURI prescriptions (prescribers 616, 618, 720) and due to an error in the coding of prescriber (prescribers 101, 102).

Appendix Table 5. Different types of medicines used by individual prescribers for AURI

Prescriber	Clinic	Sex	Age	AURI rx	Only abx		Only inf		Only TCM		Abx plus inf		Abx plus TCM		Inf plus TCM		Abx plus inf plus TCM		Only other drugs		Only abx/ only inf
				n	n	%	n	%	n	%	n	%	n	%	n	%	n	%	n	%	Ratio
101	BFZG	2	46	377	19	5.0	26	6.9	29	7.7	70	18.6	65	17.2	71	18.8	93	24.7	4	1.1	0.7
102	BFZG	1	52	56	0	0.0	30	53.6	17	30.4	5	8.9	4	7.1	0	0	0	0	0	0	0
203	CS	1	59	167	13	7.8	42	25.1	13	7.8	61	36.5	8	4.8	13	7.8	11	6.6	6	3.6	0.3
204	CS	1	46	73	30	41.1	9	12.3	2	2.7	19	26	4	5.5	3	4.1	3	4.1	3	4.1	3.3
205	CS	2	50	111	17	15.3	18	16.2	8	7.2	33	29.7	17	15.3	5	4.5	10	9	3	2.7	0.9
206	CS	1	63	173	34	19.7	29	16.8	9	5.2	69	39.9	19	11	3	1.7	8	4.6	2	1.2	1.2
307	DS	2	40	520	92	17.7	167	32.1	118	22.7	41	7.9	35	6.7	17	3.3	4	0.8	46	8.8	0.6
308	DS	1	67	217	54	24.9	62	28.6	33	15.2	10	4.6	19	8.8	3	1.4	3	1.4	33	15.2	0.9
409	FJZZ	1	63	134	46	34.3	16	11.9	7	5.2	22	16.4	18	13.4	4	3	13	9.7	8	6	2.9
410	FJZZ	1	40	199	73	36.7	20	10.1	18	9.0	36	18.1	22	11.1	9	4.5	15	7.5	6	3	3.7
411	FJZZ	1	43	124	35	28.2	15	12.1	11	8.9	19	15.3	15	12.1	11	8.9	16	12.9	2	1.6	2.3
412	FJZZ	1	60	271	92	33.9	18	6.6	13	4.8	82	30.3	31	11.4	8	3	26	9.6	1	0.4	5.1
513	QD	1	46	117	79	67.5	8	6.8	1	0.9	9	7.7	12	10.3	0	0	3	2.6	5	4.3	9.9
514	QD	1	52	578	264	45.7	86	14.9	65	11.2	100	17.3	37	6.4	13	2.2	6	1	7	1.2	3.1
515	QD	1	65	181	58	32.0	13	7.2	7	3.9	40	22.1	23	12.7	16	8.8	14	7.7	10	5.5	4.5
616	XJG	1	63	19	3	15.8	5	26.3	6	31.6	1	5.3	1	5.3	1	5.3	0	0	2	10.5	0.6
617	XJG	1	39	415	156	37.6	83	20.0	42	10.1	55	13.3	30	7.2	11	2.7	24	5.8	14	3.4	1.9
618	XJG	1	37	12	4	33.3	3	25.0	0	0.0	3	25	1	8.3	0	0	0	0	1	8.3	1.3
719	YJ	1	42	386	83	21.5	61	15.8	19	4.9	42	10.9	50	13	48	12.4	74	19.2	9	2.3	1.4
720	YJ	1	63	3	1	33.3	1	33.3	0	0.0	0	0	0	0	1	33.3	0	0	0	0	1
721	YJ	1	36	408	94	23.0	41	10.0	34	8.3	62	15.2	52	12.7	31	7.6	68	16.7	26	6.4	2.3
822	YJZZ	1	68	378	128	33.9	46	12.2	36	9.5	52	13.8	34	9	49	13	12	3.2	21	5.6	2.8
823	YJZZ	2	55	258	59	22.9	49	19.0	25	9.7	43	16.7	18	7	38	14.7	11	4.3	15	5.8	1.2

Footnote: rx = prescriptions; abx = antibiotic; inf = analgesic or anti-inflammatory medicines (any of the following: aspirin/acetylsalicylic acid, ibuprofen, paracetamol or diclofenac); TCM = traditional Chinese medicine; 1 = male; 2 = female. Rows in grey are not included in the individual prescriber-level analyses in the main text due to contributing <50 AURI prescriptions (prescribers 616, 618, 720) and due to an error in the coding of prescriber (prescribers 101, 102).
