# Supplementary material for: Variations in antibiotic prescribing among village doctors in a rural region of Shandong province, China: a cross-sectional analysis of prescriptions
Source: BMJ Open. 2020 Jun 1;10(6):e036703. doi: 10.1136/bmjopen-2019-036703 (PMC7265041; doi:10.1136/bmjopen-2019-036703)
Supplement: Author's manuscript [file bmjopen-2019-036703.draft_revisions.pdf]

BMJ Open

BMJ Open is committed to open peer review. As part of this commitment we make the peer review history of every article we publish publicly available.

When an article is published we post the peer reviewers' comments and the authors' responses online. We also post the versions of the paper that were used during peer review. These are the versions that the peer review comments apply to.

The versions of the paper that follow are the versions that were submitted during the peer review process. They are not the versions of record or the final published versions. They should not be cited or distributed as the published version of this manuscript.

BMJ Open is an open access journal and the full, final, typeset and author-corrected version of record of the manuscript is available on our site with no access controls, subscription charges or pay-per-view fees (http://bmjopen.bmj.com).

If you have any questions on BMJ Open's open peer review process please email info.bmjopen@bmj.com

BMJ Open

Variations in antibiotic prescribing among village doctors in rural Shandong province, China: a cross-sectional analysis of prescriptions

Journal:	*BMJ Open*
Manuscript ID	bmjopen-2019-036703
Article Type:	Original research
Date Submitted by the Author:	28-Dec-2019
Complete List of Authors:	Dyar, Oliver; Karolinska Institutet, Department of Global Public Health<br>Ding, Yang; Shandong University, School of Health Care Management<br>Yin, Jia; Shandong University, School of Health Care Management<br>Sun, Qiang; Shandong University, School of Health Care Management<br>Lundborg, Cecilia; Karolinska Institutet, Department of Global Public Health
Keywords:	PRIMARY CARE, Public health < INFECTIOUS DISEASES, Quality in health care < HEALTH SERVICES ADMINISTRATION & MANAGEMENT, Respiratory infections < THORACIC MEDICINE

SCHOLARONE™
Manuscripts

I, the Submitting Author has the right to grant and does grant on behalf of all authors of the Work (as defined in the below author licence), an exclusive licence and/or a non-exclusive licence for contributions from authors who are: i) UK Crown employees; ii) where BMJ has agreed a CC-BY licence shall apply, and/or iii) in accordance with the terms applicable for US Federal Government officers or employees acting as part of their official duties; on a worldwide, perpetual, irrevocable, royalty-free basis to BMJ Publishing Group Ltd ("BMJ") its licensees and where the relevant Journal is co-owned by BMJ to the co-owners of the Journal, to publish the Work in this journal and any other BMJ products and to exploit all rights, as set out in our licence.

The Submitting Author accepts and understands that any supply made under these terms is made by BMJ to the Submitting Author unless you are acting as an employee on behalf of your employer or a postgraduate student of an affiliated institution which is paying any applicable article publishing charge ("APC") for Open Access articles. Where the Submitting Author wishes to make the Work available on an Open Access basis (and intends to pay the relevant APC), the terms of reuse of such Open Access shall be governed by a Creative Commons licence – details of these licences and which Creative Commons licence will apply to this Work are set out in our licence referred to above.

Other than as permitted in any relevant BMJ Author's Self Archiving Policies, I confirm this Work has not been accepted for publication elsewhere, is not being considered for publication elsewhere and does not duplicate material already published. I confirm all authors consent to publication of this Work and authorise the granting of this licence.

For peer review only

TITLE: Variations in antibiotic prescribing among village doctors in rural Shandong province, China: a cross-sectional analysis of prescriptions

Oliver J. Dyar[a]*, Yang Ding[b]*, Yin Jia[b], Sun Qiang[b+], Cecilia Stålsby Lundborg[a]

[a]Department of Global Public Health, Karolinska Institutet, Stockholm, Sweden

[b]School of Health Care Management, Shandong University, Jinan, Shandong, China; Key Laboratory of Health Economics and Policy Research, NHC, Shandong University, Jinan, Shandong, China; Center for Health Management and Policy Research, Shandong University, Jinan, Shandong, China

*Contributed equally

[+]Corresponding author: School of Health Care Management, Shandong University, Jinan, Shandong, China. Tel: +86-531-88382376; Fax: +86-531-88382693; E-mail: qiangs@sdu.edu.cn.

Word count: 3413

For peer review only

ABSTRACT

Objectives: To assess variation in antibiotic prescribing practices among village doctors in rural Shandong province, China.

Design, setting and participants: Prescriptions were collected over a 2.5 year period from eight primary care village clinics staffed by 23 doctors located around a single town in rural Shandong province. A target of 60 prescriptions per clinic per month were sampled from an average total of around 300. Prescriptions were analysed at both aggregate and individual-prescriber levels, with a focus on diagnoses of likely viral acute upper respiratory tract infections (AURI), defined as ICD-10 codes J00 and J06.9.

Main outcome measures: Proportions of prescriptions for AURIs containing i) at least one antibiotic, ii) multiple antibiotics, iii) at least one parenteral antibiotic; classes and agents of antibiotics prescribed.

Results: In total 14471 prescriptions from 23 prescribers were included, of which 5833 (40.3%) contained at least one antibiotic. Nearly two-thirds 62.5% (3237/5177) of likely viral AURI prescriptions contained an antibiotic, accounting for 55.5% (3237/5833) of all antibiotic-containing prescriptions. There was wide variation at the individual-level in antibiotic prescribing rates for AURIs (33.1% to 88.0%), as well multiple antibiotic prescribing rates (1.3% to 60.2%) and parenteral antibiotic prescribing rates (3.2% to 62.1%). Each doctor prescribed between 11 and 21 unique agents for AURIs, including many broad-spectrum antibiotics. Doctors in the highest quartile for antibiotic prescribing rates for AURI also had a trend towards higher antibiotic prescribing rates for potentially bacterial upper respiratory tract infections (pharyngitis, tonsillitis, laryngopharyngitis) (92.7% vs. 72.4%, p = 0.08) compared with doctors in the lowest quartile.

Conclusions: All village doctors over-used antibiotics for respiratory tract infections. Variations in individual prescriber practices are significant even in a small homogenous setting, and should be accounted for when developing targets and interventions to improve antibiotic use.

KEYWORDS

Primary care; Public health; Quality in health care; Respiratory infections

ARTICLE SUMMARY

Strengths and limitations of this study

- We investigated whether significant variations exist in antibiotic prescribing patterns among village doctors working in a small, homogenous setting in rural China
- We included over 14000 prescriptions sampled across a 2.5 year time period
- Uniquely, we analysed prescriptions according to clinical diagnosis, focussing on acute upper respiratory tract infections, and at the level of individual prescribers
- We could not verify diagnoses clinically, and so variations between the prescribers in terms of diagnostic ability could not be assessed

For peer review only

BACKGROUND

Irrational antibiotic use is an important and modifiable driver of antibiotic resistance.[1] Understanding specific patterns of unnecessary or inappropriate antibiotic use is essential to designing context-adapted, targeted interventions to improve antibiotic use, such as education focussing on common irrational practices within a region or institution.[2] Understanding variations in antibiotic prescribing patterns at the prescriber-level can be valuable both for shedding light on facilitators and barriers to rational antibiotic use, as well as for enabling individualised feedback on prescribing practices.[3,4]

Previous studies in China strongly indicate that there is widespread antibiotic overuse in outpatient clinics in rural areas, particularly for respiratory tract infections.[5–8] These studies have not been capable of investigating the extent of variability in practices that exist, due to two common limitations: first, most studies have relied on highly aggregated data (e.g. at the level of an institution or county); second, some studies have not been able to couple antibiotic prescriptions with clinical diagnoses at the level of the individual prescription. Recent studies in other countries have highlighted the value of such finer-grained analyses; for example, Pouwels *et al.* found that variability in prescribing patterns for antibiotics at primary care centres in the UK could not be adequately explained by variations in patient consultation rate or pre-existing co-morbidities,[9] i.e. individual prescriber behaviours are an important cause of irrational antibiotic use, and so need to be addressed.[10]

We investigated patterns of antibiotic prescriptions at the individual prescriber-level in eight village clinics in rural Shandong province, China, over a two-and-a-half-year time period. Village clinics are the first place that most rural residents seek healthcare. These are staffed by village doctors who typically have a high school level education and additional vocation-related training; many of the older village doctors were trained under the barefoot doctor programme.[11] Our aims were to assess if there was overall evidence of irrational antibiotic use, and whether there was significant variability between the practices of individual prescribers in this small, homogenous region. Our study forms part of a large cross-sectoral research programme on antibiotic consumption and resistance involving Chinese and Swedish researchers, the Sino-Swedish Integrated Multi-sectoral Partnership for Antibiotic Resistance Containment (IMPACT).[12]

MATERIALS AND METHODS

Study design and setting

We conducted a retrospective analysis of outpatient prescriptions from January 2015 to July 2017 at eight village clinics clustered around a single town in Z County, Shandong province. This county is in middle level in Shandong province in terms of economics, health indicators and population size. As previously described in the full study protocol,[12] the county and town were first selected based on the requirements and design of the entire IMPACT research programme. Twelve villages were then purposively selected around the town from among seventeen that had at least one hundred households. These twelve villages are served by eight village clinics located in the villages and are attended by 23 doctors who do not move between clinics.

Data collection

The prescriptions of all village clinics in Z county are included in an e-prescription system that

For peer review only

records for each visit the patients' name, sex, age, date of visit, diagnosis, medicines prescribed, total cost for the visit and name of the doctor. For each village clinic a target of 60 prescriptions per month (from an average total of around 300) were collected using a random sampling method in which every fifth consecutive prescription was selected from a randomly generated starting number from 1 to 10. The prescription details were exported one-by-one from the e-prescription system in XPS format and entered into a Microsoft Excel database. In addition, in July 2015 the doctors completed a short face-to-face questionnaire concerning their demographic information (gender, age, number of years working).

Data management and analyses

Prescriptions from one doctor who did not complete the questionnaire were removed (55 in total). Data were then anonymised with patient names being removed and each doctor assigned a three-digit code representing the village clinic at which they worked (1XX to 8XX) and a unique number (X01 to X23). All unique diagnoses were translated from Chinese into English and coded where possible according to the International Classification of Diseases, 10th Revision (ICD-10) by an independent researcher. A category of *likely viral acute upper respiratory tract infections* (AURI) was created by grouping diagnoses of J00 (acute nasopharyngitis [common cold]) and J06.9 (acute upper respiratory infection, unspecified). Prescriber-level analyses were limited to doctors with ≥50 AURI prescriptions during the study period. We identified an error in the coding of the doctor responsible for the prescription from village clinic 1, so all 433 prescriptions from this clinic were excluded from prescriber-level analyses. All prescribed antibiotics prescribed were coded using the Anatomical Therapeutic Chemical (ATC) classification system[13] and categorised by classes and substances. Prescribed medicines were also coded as: "Analgesic or anti-inflammatory medicines" (aspirin/acetylsalicylic acid, ibuprofen, paracetamol or diclofenac); "traditional Chinese medicines"; or "other".

For each prescriber, antibiotic prescribing was analysed using three indicators: a) antibiotic prescribing rate (APR) = number of prescriptions that included at least one antibiotic / total number of prescriptions x 100%, b) multi-antibiotic prescribing rate (MPR) = number of prescriptions that included at least two antibiotics with different ATC codes / number of prescriptions that included at least one antibiotic x 100%, c) parenteral antibiotic prescribing rate (PAPR) = number of prescriptions that included at least one parenteral antibiotic / number of prescriptions that included at least one antibiotic x 100%. Comparisons were also made against antibiotic prescribing quality indicators developed in Europe[14] for a subset of clinical diagnoses and patient ages.

All analyses were conducted in Microsoft Excel and SPSS. Descriptive statistics were calculated and comparisons were made using χ^2 tests and Pearson correlation. Linear regression was used to assess trends in the APR for AURI. Statistical significance was set at $p < 0.05$ for all tests.

Patient and public involvement

Patients or the public were not involved in the design, conduct, reporting, or dissemination plans of this specific study, but have been involved in the dissemination plans for results of the overall IMPACT research programme.

Ethics

Informed consent was obtained from all village doctors. Ethical approval was obtained from the First Affiliated Hospital, College of Medicine, Zhejiang University, China (reference numbers 2015#185 and 2015#283).

RESULTS

Overview of prescriptions and diagnoses

In total 14471 prescriptions from 23 prescribers from January 2015 to July 2017 were included in the analyses. This included an average of 58 prescriptions per month per village clinic (mean range from 47 to 62). There was a total of 222 diagnoses in Chinese, with 20 diagnoses accounted for nearly 90% of all prescriptions (12811/14471). The five most common diagnoses overall were: J00 (acute nasopharyngitis [common cold], 34.1%, 4938/14471), I10 (Essential hypertension, 13.6%, 1966/14471), K29.7 (Gastritis, unspecified, 5.9%, 856/14471), M13.9 (Arthritis, unspecified, 5.9%, 955/14471) and I25.1 (Atherosclerotic heart disease, 3.2%, 470/14471). Overall, 40.3% of the prescriptions (5833/14471) contained at least one antibiotic.

Table 1 summarises the infection-related diagnoses that had a minimum of ten prescriptions during the study period. Overall, respiratory tract infections accounted for 68.4% (3991/5833) and gastrointestinal conditions for 14.0% (815/5833) of all prescriptions containing at least one antibiotic. A total of 5177 prescriptions were categorised as *likely viral acute upper respiratory tract infections* (AURI), and 62.5% (3237/5177) of these prescriptions contained at least one antibiotic, accounting for 55.5% (3237/5833) of all antibiotic use. Fifteen percent (491/3237) of AURI antibiotic prescriptions included two or more antibiotics and 24.3% (785/3237) included at least one parenteral antibiotic.

Table 1. Infection-related diagnoses and antibiotic prescription rates

Body system (no. prescriptions)	Diagnosis	ICD-10 code	No. of prescriptions containing an antibiotic / total no. prescriptions	APR (%)
Respiratory (6183)	Acute nasopharyngitis [common cold]⁺	J00	3034/4938	61.4
	Bronchitis*	J40	408/522	78.2
	Acute upper respiratory infection⁺	J06.9	203/239	84.9
	Acute pharyngitis	J02.9	160/190	84.2
	Acute tonsillitis	J03.9	68/73	93.2
	Rhinitis*	NA	31/67	46.3
	Cough	NA	23/65	35.4
	Pneumonia	J18	40/51	78.4
	Bronchopneumonia	J18.0	15/19	78.9
	Acute laryngopharyngitis	J06.0	8/14	57.1
Gastrointestinal (1642)	Gastritis	K29.7	258/856	30.1
	Gastroenteritis	A09.9	496/717	69.2
	Diarrhoea	NA	61/69	88.4
Dental (222)	Chronic periodontitis	K05.3	100/131	76.3
	Gingivitis and periodontal diseases	K05	61/72	84.7
	Pulpitis	K04.0	19/19	100.0
Urogenital (84)	Inflammatory disease of prostate	N41.9	23/45	51.1
	Urethritis and urethral syndrome	N34	23/24	95.8

For peer review only

	Urinary tract infection	N39.0	15/15	100.0
Eye (101)	Conjunctivitis	H10	9/101	8.9
Ear (11)	Otitis media	H66.9	7/11	63.6

Table footnote: *Not specified as acute or chronic; +Categorised as likely viral acute upper respiratory tract infection; APR = antibiotic prescription rate.

Medicines prescribed for AURI

Figure 1 shows the combinations of different medicine types (antibiotics, anti-inflammatories or analgesics, and traditional Chinese medicines) prescribed for AURI. Approximately half of all prescriptions with AURI (54.0%, 2795/5177) contained only one medicine type, a third (33.7%, 1744/5177) contained two medicine types, and 8% (414/5177) contained all three medicine types.

[Insert Figure 1]

Sixteen classes of antibiotics and 27 antibiotic agents were prescribed for AURI (Appendix Table 1). The three most common classes of antibiotics prescribed were: J01CA (Penicillins with Extended Spectrum, 31.6% of prescriptions), J01DB (First-Generation Cephalosporins, 18.6% of prescriptions), J01FA (Macrolides, 15.7% of prescriptions). The three most common agents of antibiotics prescribed for AURI were J01CA04 (Amoxicillin, 31.4% of prescriptions), J01MA12 (Levofloxacin, 10.3% of prescriptions), J01DB01 (Cefalexin, 9.9% of prescriptions).

Comparison with European quality indicators

Table 2 shows antibiotic prescribing rates and types of antibiotic for a variety of respiratory tract infections in our database based on previously published European recommendations.[14]

Table 2. **Antibiotic prescribing quality indicators for selected diagnoses**

Diagnosis	Patient age group (years)	APR n/N, %	Class of antibiotics		
			**J01M** n/N, %	**J01CE** n/N, %	**J01AA or J01CA** n/N, %
AURI	≥2	3236/5172, 62.6	436/3236, 13.5	49/3236, 1.5	
Target		*0-20%*	*0-5%*	*80-100%*	NA
Tonsillitis	≥2	68/73, 93.2	15/68, 22.1	2/68, 2.9	
Target		*0-20%*	*0-5%*	*80-100%*	NA
Pneumonia	18 to 65	22/30, 73.3	11/22, 50.0		2/22, 12.8
Target		*90-100%*	*0-5%*	NA	*80-100%*

Table footnote: APR = antibiotic prescribing rate; targets are as previously published,[14] except for "NA" which indicates that no target was included; J01M is the ATC code for Quinolone Antibacterials, J01CE is the ATC code for Beta-Lactamase Sensitive Penicillins, J01AA is the ATC code for Tetracyclines, J01CA is the ATC code for Penicillins with Extended Spectrum.

Prescriber demographics

Table 3 summarises prescribers' demographic information and antibiotic prescribing rates for patients with AURI. The median age of the prescribers was 51 years old, and the median years of working experience was 31. Male prescribers were more likely to prescribe antibiotics for their

For peer review only

patients than female prescribers (67.7% versus 42.7%, $p < 0.01$). Male prescribers were slightly more lightly to prescribe antibiotics for female patients than for male patients (69.5% versus 66.3%, $p = 0.04$), but no difference was seen among female prescribers (41.9% versus 43.4%, $p = 0.65$).

Table 3. Prescriber demographics and antibiotic prescribing rates for AURIs

Prescriber	No. of prescribers		AURI prescriptions			
	n	%	n	APR	MPR	PAPR
Total			*4710*	*63.0*	*15.7*	*26.0*
Gender						
Male	15	83.3	3821	67.7	17.1	28.4
Female	3	16.7	889	42.7	5.8	9.7
Age						
36~39	2	11.1	823	65.7	18.7	26.1
40~49	6	33.3	1419	57.2	24.5	30.6
50~59	4	22.2	1114	63.6	8.2	27.4
60~68	6	33.3	1354	67.0	11.8	20.7
Education*						
Junior high school	3	16.7	962	60.9	9.7	34.0
Senior high school	15	83.3	3748	63.5	17.1	24.0
Working years						
14~19	5	27.8	1666	56.7	17.5	26.8
20~29	4	22.2	687	70.6	28.0	29.1
30~39	2	11.1	836	64.4	10.0	34.6
40~48	7	38.9	1521	65.7	11.0	19.1

Table footnote: APR = antibiotic prescribing rate; MPR = multiple antibiotic prescribing rate; PAPR = parenteral antibiotic prescribing rate. *Education: Junior high school only is equivalent to completing 9 years of full-time education; senior high school is equivalent to completing 12 years of full-time education.

For peer review only

Variations at the prescriber-level

Overall, individual prescribers' AURI antibiotic prescribing rates were highly correlated between 2015 and 2016 ($r = 0.6455$). The mean absolute individual variation in AURI antibiotic prescribing rates between 2015 and 2016 was +/-13.1% (standard deviation = 6.7%). Eleven prescribers had lower AURI antibiotic prescribing rates in 2016 compared with 2015, and six had higher antibiotic prescribing rates in 2016 (Appendix Table 2).

Amoxicillin was the most commonly prescribed agent for AURI for 13/18 prescribers; the other agents that were the single most commonly prescribed for individual prescribers included levofloxacin (2 doctors), amikacin (1), lincomycin (1) and azithromycin (1). For each prescriber, their three most commonly prescribed antibiotic agents accounted for a mean of 64.4% (range 45.3% to 86.4%) of all the antibiotics they prescribed for AURI (Appendix Table 3).

Figure 2 and Appendix Table 4 illustrate the individual variations between the eighteen doctors for AURI prescriptions. There was wide variation in the antibiotic prescribing rate (33.1% to 88.0%), multiple antibiotic prescribing rate (1.3% to 60.2%) and parenteral antibiotic prescribing rate (3.2%

to 62.1%). The APR was positively correlated with MPR (r = 0.472, p = 0.05) and PAPR (r = 0.54, p = 0.02), but there was no association between APR and the prescription of at least one analgesic or anti-inflammatory medicine (r = -0.19, p = 0.46). The MPR was also positively correlated with PAPR (r = 0.77, p < 0.01).

[Insert Figure 2]

Figure 3 and Appendix Table 5 illustrate the different types of medicines used for AURI prescriptions by 18 prescribers. The most common choice for prescribers (14/18) was to use only an antibiotic; the most common choice for the four remaining prescribers was to use only analgesic or anti-inflammatory medicines. All prescribers prescribed all three types of medicines for patients with a diagnosis of AURI on at least one occasion.

[Insert Figure 3]

Comparisons between high and low antibiotic prescribers

Table 4 shows comparisons for selected indicators between the four prescribers with the highest antibiotic prescribing rate for AURI (513, 412, 204 and 206) and the four prescribers with the lowest APR for AURI (203, 823, 308, 307).

Table 4. Variation between high and low AURI antibiotic prescribing rate groups

Indicator	High AURI APR group (%)	Low AURI APR group (%)	χ^2 *value*	*P value*
Mean APR for AURI	81.3	44.8	273.309	<0.001
Mean MPR for AURI	23.4	6.2	36.058	<0.001
Mean PAPR for AURI	33.6	10.7	44.570	<0.001
Mean prescribing rate of only antibiotics for AURIs	40.6	18.3	72.877	<0.001
Mean prescribing rate of only analgesics/anti-inflammatory medicines for AURI	10.6	26.2	74.256	<0.001
Mean APR for URTI with potential bacterial causes*	98.2	84.0	3.015	0.082
Mean APR for gastritis, gastroenteritis and diarrhoea	88.8	76.2	0.617	0.432

Table footnote: High AURI APR group contain the four prescribers with the highest antibiotic prescribing rate for AURI, and low AURI APR group contain the four prescribers with the lower antibiotic prescribing rate for AURI.

APR = antibiotic prescribing rate; MPR = multi-antibiotic prescribing rate; PAPR = parenteral antibiotic prescribing rate;

*URTI with potential bacterial causes = prescriptions containing a diagnosis of pharyngitis, tonsillitis or laryngopharyngitis.

DISCUSSION

We retrospectively investigated patterns of antibiotic prescriptions over a two-and-a-half-year period in village clinics in rural Shandong province in order to assess evidence of irrational antibiotic use and variability in prescribing practices at the level of the individual prescriber.

Consultation patterns and overall antibiotic use

The patterns of diagnoses on the prescriptions in our study are broadly similar to those in previous

For peer review only

studies conducted in rural China,[5,15] with respiratory tract infections accounting for a large proportion of patient visits. Diagnoses of dental conditions and gastro-enteritis and related symptoms were more common than in primary care consultations in high income countries, whereas urinary tract infections and skin- and soft-tissue infections accounted for a far smaller proportion of patient visits. Overall, forty percent of prescriptions contained at least one antibiotic. This antibiotic prescription rate is considerably higher than the government's target from 2012 of fewer than 20% outpatient prescriptions (for secondary level hospitals),[16,17] but is lower than previously published studies in China which tend to be around 50%.[5,7,15,18,19]

Antibiotic use for respiratory tract infections

Although the antibiotic prescription rate is high in absolute terms, supporting clinical information is required to better understand the quality of the prescribing. We found that two-thirds of all antibiotic use occurred in patients with a diagnosis of a respiratory tract infection (RTI), and that likely viral acute upper respiratory tract infections accounted for over 80% of the total antibiotic use for RTIs. This is despite there being very little evidence of benefit of antibiotic for such AURIs, and a considerable number of strong recommendations that they should be avoided.[20,21] The antibiotic prescribing rate for AURIs of 60% is similar to previous studies in rural village clinics in Shandong province,[8] Anhui province[22] and in Western Chin [15] as well as reports from rural India,[4,23] and Malaysia,[24] but far higher than rates observed in national-level studies in Sweden (8%), Belgium (19%) and Netherlands (38%).[25]

Antibiotic prescribing quality indicators were recently developed in Europe to assess both the decision to prescribe an antibiotic in specific clinical conditions, and the actual choice of antibiotic agent.[14] All of our results lay outside of the recommended ranges. In particular, we found that the recommended J01CE antibiotics were rarely used (1.5% for AURI, 2.9% for tonsillitis), whereas J01CA penicillins were used far more frequently. This may represent a prescriber preference for broader spectrum antibiotics. Interestingly, we found that antibiotics were used less frequently than recommended for adult patients with pneumonia (73.3% compared with a target of 90-100%),[14] potentially representing underuse of antibiotics. Analgesic and anti-inflammatory medicines are potentially under-used, since they have the potential to both provide symptomatic relief and satisfy patients' expectations to gain some form of medicine from visiting the doctor; fewer than half of all patients with a diagnosis of AURI received such a medication.

Variations in antibiotic prescribing between prescribers

Understanding what variations exist in clinical care, and why, is important for developing targets as well as context-specific interventions capable of reaching these targets. It is also essential for working towards individuals having equitable access to healthcare, irrespective of where they reside.

Our study design has enabled us to investigate variability in antibiotic prescribing at the prescriber-level whilst keeping several factors relatively constant: the villages served by the clinics are limited to a small geographical region and are quite homogeneous in terms of socio-economic levels; the patient populations in the villages are likely to have similar consultation behaviours when ill, in particular for a single diagnosis such as AURI; the doctors all have at least 14 years of clinical experience and have the same medical qualifications; the village clinics are all subject to the same reimbursement systems (including zero mark-up on antibiotics) and regulations

For peer review only - http://bmjopen.bmj.com/site/about/guidelines.xhtml

concerning antibiotic use.[26,27] Given these similarities, it might be hypothesised that little variation would exist in the prescribing practices between the doctors. Our results, however, revealed substantial differences between prescribers. Patients with AURI who were seen by the high-antibiotic prescribers were twice as likely to be given a prescription containing an antibiotic, four times as likely to be given two or more antibiotics, and three times as likely to receive at least one parenteral antibiotic. There was also significant variation within the high-prescriber group, highlighting the importance of individual-level analyses: patients with AURI who visited doctors 513 and 412 were equally likely to be prescribed an antibiotic (mean APR of 87%), but those who visited doctor 513 were six times more likely to be prescribed two or more antibiotics, three times more likely to be prescribed an parenteral antibiotic, and a third less likely to be prescribed an analgesic or anti-inflammatory medicine; furthermore, the choice of antibiotic prescribed was likely to be an aminoglycoside, a third generation cephalosporin or a fluoroquinolone, compared with amoxicillin, a first-generation cephalosporin, or a macrolide. Furthermore, a single doctor was responsible for a quarter of all amikacin use during the study period. Such extreme variation is important for individual patients in terms of risks of developing antibiotic-related complications and the impact of antibiotic use on their normal flora.[28] This ethical component should not be understated, and there is a growing trend for antibiotic stewardship to be viewed in terms of patient safety and broader responsibility.[29,30]

Very few published studies have assessed variations between prescribers concerning antibiotic use, with most studies analysing aggregated data and presenting averages. Akkerman *et al* investigated antibiotic prescribing rate for upper respiratory tract infections among 84 primary care practitioners in the Netherlands, finding that this ranged from 15 to 27%.[31] Grover *et al* examined antibiotic prescribing for RTIs at a single outpatient practice in the US and found variations depending on the provider type from 55% (residents) to 84% (nurse practitioners).[32] A tempting justification against conducting such individual-level analyses is that it is difficult to account for the variation in patient case mix. This does indeed limit the ability to make comparisons, particularly between studies conducted in settings in which patients have very different consultation rates and co-morbidities, or in studies that look at overall antibiotic prescription rates rather than at a single diagnosis. However, there is growing evidence that differences in patient case mix may not explain a large amount of variability in prescribing patterns for antibiotics.[9] Furthermore, ignoring variations between individual prescribers causes us to subtly obscure the role (or even responsibility) of the individual decision-maker. Interestingly, the doctors in our study each used between 11 and 21 different antibiotic agents for patients with AURI, demonstrating the heterogeneity even within an individual prescriber.

Future studies should explore why such variations exist, as well as what the consequences are for efforts to improve antibiotic use. For example, qualitative studies with individual prescribers could help understand why some doctors already have very low rates of parenteral and multi-antibiotic use, and why prescribers have used such a wide range of antibiotic agents for patients with a single diagnosis. It is possible that structural factors such medicine shortages might contribute to changes in antibiotics prescribed. We did find that high antibiotic AURI prescribers were more likely than low AURI prescribers to prescribe antibiotics for potentially bacterial URTIs. One explanation is that these prescribers are more risk averse;[33] an alternative is that these prescribers are more willing than others to follow patient demand, particularly if this is generally high. Future studies could also explore the influence of peers within clinics; our results suggest

there is some clustering of behaviours within certain village clinics.

Methodological considerations

Our study has several strengths, including the long length of data collection and the high number of prescriptions included by individual prescribers. Furthermore, all patients that visit a doctor at a village clinic receive prescribed medicines. A common limitation of studies using prescription data is the inability to verify diagnoses. To restrict the impact of this limitation we restricted the individual-level analyses to AURI diagnoses, but we cannot guarantee that misclassifications did not occur, particularly given there is currently no incentive for doctors to correctly classify diagnoses. AURIs accounted on average for 86% of respiratory tract infection diagnoses by individual doctors, but this ranged from 58% to 100% which strongly suggests that there were variations in how doctors classified different infections, and this warrants further investigation, particularly if diagnoses-based antibiotic prescribing targets are ever introduced. Indeed, a recent study concluded that deficits in diagnostic knowledge are a major driver of unnecessary antibiotic prescriptions in clinics in rural China.[34] Additional limitations include an inability to account for variations in the workload of individual doctors, and to control for patient co-morbidities.

Conclusion

Together our results provide evidence that even in a small homogenous setting in rural China, variations in individual prescriber practices are significant, and they need to be accounted for in the development of targets and interventions to improve antibiotic use. We suspect this finding is likely to be generalizable to other areas in rural China, where the healthcare system is very similar in terms of structure, regulations and staffing. We show that there is a need for all prescribers to reduce their antibiotic prescribing rate for AURIs, but the extent of this reduction varies widely between prescribers. For some prescribers, reducing the proportion of prescriptions that contain fluoroquinolones and third-generation cephalosporins may be more important goals, both for their patients and for public health. Finally, it is possible that interventions that incorporate individual-level feedback will have a more directed and effective impact on improving antibiotic use. A promising recent example in Anhui province, China, was able to provide “just-in-time” individualised information and feedback to prescribers in village clinics.[35]

Acknowledgements

We acknowledge the support of the local Centre for Disease Control and Prevention in coordinating this study, as well as all students who contributed to the data collection. We would also like to thank all residents who agreed to participate in the IMPACT research programme. We are grateful for the efforts of the IMPACT-consortium: www.folkhalsomyndigheten.se/impact/.

Competing interests

None to declare.

Funding

This work was supported by the Swedish Research Council (grant number D0879801) and National Natural Science Foundation of China (grant number 81361138021).

Author contributions

All authors were involved in the design of the study. YD and YJ participated in the data collection.

For peer review only

OJD, YD conducted the analyses and drafted the manuscript, with YJ, SQ and CSL giving critical input. All authors read and approved the final manuscript.

Data sharing statement

The data sets generated and analysed during the current study are available on reasonable request from the corresponding author.

REFERENCES

1 van de Sande-Bruinsma N, Grundmann H, Verloo D, *et al.* Antimicrobial drug use and resistance in Europe. *Emerg Infect Dis* 2008;14:1722–30.

2 Davey P, Marwick CA, Scott CL, *et al.* Interventions to improve antibiotic prescribing practices for hospital inpatients. *Cochrane Database Syst Rev* 2017;2:CD003543.

3 Tamma PD, Cosgrove SE. Antimicrobial stewardship. *Infect Dis Clin North Am* 2011;25:245–60.

4 Dehn Lunn A. Reducing inappropriate antibiotic prescribing in upper respiratory tract infection in a primary care setting in Kolkata, India. *BMJ Open Qual* 2018;7:e000217.

5 Wang J, Wang P, Wang X, *et al.* Use and prescription of antibiotics in primary health care settings in china. *JAMA Intern Med* 2014;174:1914–20.

6 Li J, Song X, Yang T, *et al.* A Systematic Review of Antibiotic Prescription Associated With Upper Respiratory Tract Infections in China. *Medicine (Baltimore)* 2016;95:e3587.

7 Jiang Q, Yu BN, Ying G, *et al.* Outpatient prescription practices in rural township health centers in Sichuan Province, China. *BMC Health Serv Res* 2012;12:324.

8 Sun Q, Dyar OJ, Zhao L, *et al.* Overuse of antibiotics for the common cold – attitudes and behaviors among doctors in rural areas of Shandong Province, China. *BMC Pharmacol Toxicol* 2015;16:6.

9 Pouwels KB, Dolk FCK, Smith DRM, *et al.* Explaining variation in antibiotic prescribing between general practices in the UK. *J Antimicrob Chemother* 2018;73:ii27–35.

10 Stålsby Lundborg C, Tamhankar AJ. Understanding and changing human behaviour--antibiotic mainstreaming as an approach to facilitate modification of provider and consumer behaviour. *Ups J Med Sci* 2014;119:125–33.

11 Asia Pacific Observatory on Health Systems and Policies. People's Republic of China Health System Review. Manila: WHO Regional Office for the Western Pacific 2015.

12 Sun Q, Wang Y, Hulth A, *et al.* Study protocol for One Health data collections, analyses and intervention of the Sino-Swedish integrated multisectoral partnership for antibiotic resistance containment (IMPACT). *BMJ Open* 2018;8:e017832.

13 WHO Collaborating Centre for Drug Statistics Methodology. ATC classification index with DDDs, 2018. https://www.whocc.no/atc_ddd_index/ (accessed 1 August 2018).

14 Adriaenssens N, Coenen S, Tonkin-Crine S, *et al.* European Surveillance of Antimicrobial Consumption (ESAC): disease-specific quality indicators for outpatient antibiotic prescribing. *BMJ Qual Saf* 2011;20:764–72.

15 Dong L, Yan H, Wang D. Antibiotic prescribing patterns in village health clinics across 10 provinces of Western China. *J Antimicrob Chemother* 2008;62:410–5.

16 Xiao Y, Li L. The actions of China in containing antimicrobial resistance. In: AMR Control 2015. 2015:46–53. http://www.globalhealthdynamics.co.uk/wp-content/uploads/2015/05/07_Xiao-Li.pdf (accessed 1 Dec 2019).

For peer review only

17 National Health Commission of the People's Republic of China. Further Strengthening the Management of Clinical Application of Antibacterial Medicines. 2015. http://www.nhc.gov.cn/xxgk/pages/viewdocument.jsp?dispatchDate=&staticUrl=/yzygj/s3593/201508/f0fdf1f52df14b87aa97be53819f1036.shtml (accessed 1 Dec 2019)

18 Yin X, Song F, Gong Y, *et al.* A systematic review of antibiotic utilization in China. *J Antimicrob Chemother* 2013;68:2445–52.

19 Liu C, Liu C, Wang D, *et al.* Determinants of antibiotic prescribing behaviors of primary care physicians in Hubei of China: a structural equation model based on the theory of planned behavior. *Antimicrob Resist Infect Control* 2019;8:23.

20 Harris AM, Hicks LA, Qaseem A, *et al.* Appropriate Antibiotic Use for Acute Respiratory Tract Infection in Adults: Advice for High-Value Care From the American College of Physicians and the Centers for Disease Control and Prevention. *Ann Intern Med* 2016;164:425.

21 Kenealy T, Arroll B. Antibiotics for the common cold and acute purulent rhinitis. *Cochrane Database Syst Rev* 2013;:CD000247.

22 Chai J, Coope C, Cheng J, *et al.* Cross-sectional study of the use of antimicrobials following common infections by rural residents in Anhui, China. *BMJ Open* 2019;9:e024856.

23 Khare S, Purohit M, Sharma M, *et al.* Antibiotic Prescribing by Informal Healthcare Providers for Common Illnesses: A Repeated Cross-Sectional Study in Rural India. *Antibiotics* 2019;8:139.

24 Ab Rahman N, Teng CL, Sivasampu S. Antibiotic prescribing in public and private practice: a cross-sectional study in primary care clinics in Malaysia. *BMC Infect Dis* 2016;16:208.

25 Tyrstrup M, van der Velden A, Engstrom S, *et al.* Antibiotic prescribing in relation to diagnoses and consultation rates in Belgium, the Netherlands and Sweden: use of European quality indicators. *Scand J Prim Health Care* 2017;35:10–8.

26 Yi H, Miller G, Zhang L, *et al.* Intended and unintended consequences of China's zero markup drug policy. *Health Aff (Millwood)* 2015;34:1391–8.

27 Xiao Y, Li L. Legislation of clinical antibiotic use in China. *Lancet Infect Dis* 2013;13:189–91.

28 Costelloe C, Metcalfe C, Lovering A, *et al.* Effect of antibiotic prescribing in primary care on antimicrobial resistance in individual patients: systematic review and meta-analysis. *BMJ* 2010;340:c2096.

29 Tamma PD, Holmes A, Ashley ED. Antimicrobial stewardship. *Curr Opin Infect Dis* 2014;27:348–55.

30 Dyar OJ, Obua C, Chandy S, *et al.* Using antibiotics responsibly: are we there yet? *Future Microbiol* 2016;11:1057–71.

31 Akkerman AE, Kuyvenhoven MM, van der Wouden JC, *et al.* Prescribing antibiotics for respiratory tract infections by GPs: management and prescriber characteristics. *Br J Gen Pract* 2005;55:114–8.

32 Grover ML, Mookadam M, Rutkowski RH, *et al.* Acute respiratory tract infection: a practice examines its antibiotic prescribing habits. *J Fam Pract* 2012;61:330–5.

33 Deschepper R, Grigoryan L, Lundborg CS, *et al.* Are cultural dimensions relevant for explaining cross-national differences in antibiotic use in Europe? *BMC Health Serv Res* 2008;8:123.

34 Xue H, Shi Y, Huang L, *et al.* Diagnostic ability and inappropriate antibiotic prescriptions: a quasi-experimental study of primary care providers in rural China. *J Antimicrob Chemother* 2019;74:256–63.

35 Shen X, Lu M, Feng R, *et al.* Web-Based Just-in-Time Information and Feedback on Antibiotic

For peer review only

Use for Village Doctors in Rural Anhui, China: Randomized Controlled Trial. *J Med Internet Res* 2018;20:e53.

For peer review only

FIGURE LEGENDS

Figure 1. Medicine types prescribed for AURIs

Figure footnote: Analgesics or anti-inflammatories = any of the following: aspirin/acetylsalicylic acid, ibuprofen, paracetamol or diclofenac.

Figure 2. Antibiotic prescribing rates for AURIs for individual prescribers

Figure 3. Different medicine types used for AURIs by individual prescribers

For peer review only

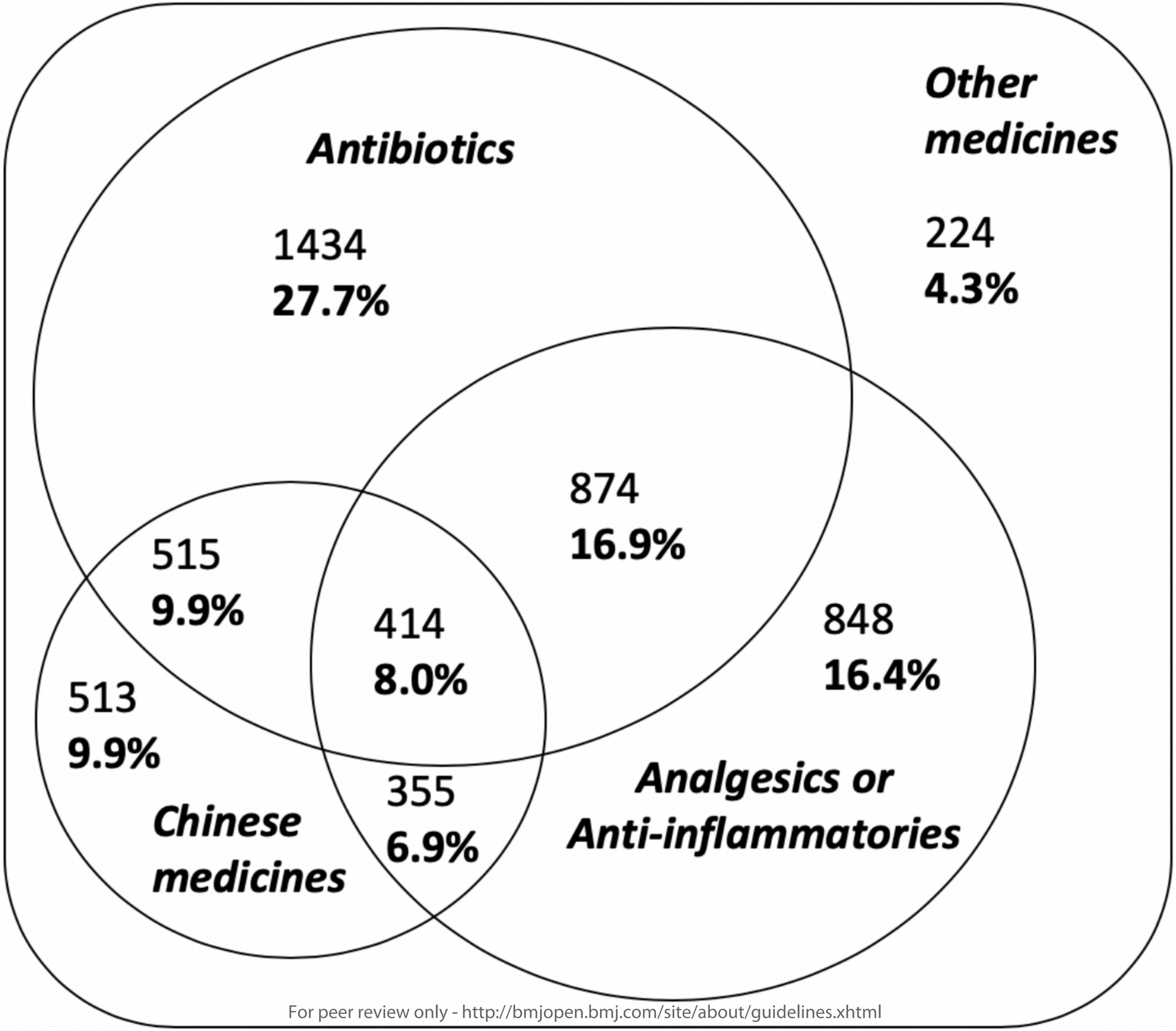
Antibiotics
Other medicines
1434
27.7%
224
4.3%
874
16.9%
515
9.9%
414
8.0%
848
16.4%
513
9.9%
355
6.9%
Chinese medicines
Analgesics or Anti-inflammatories

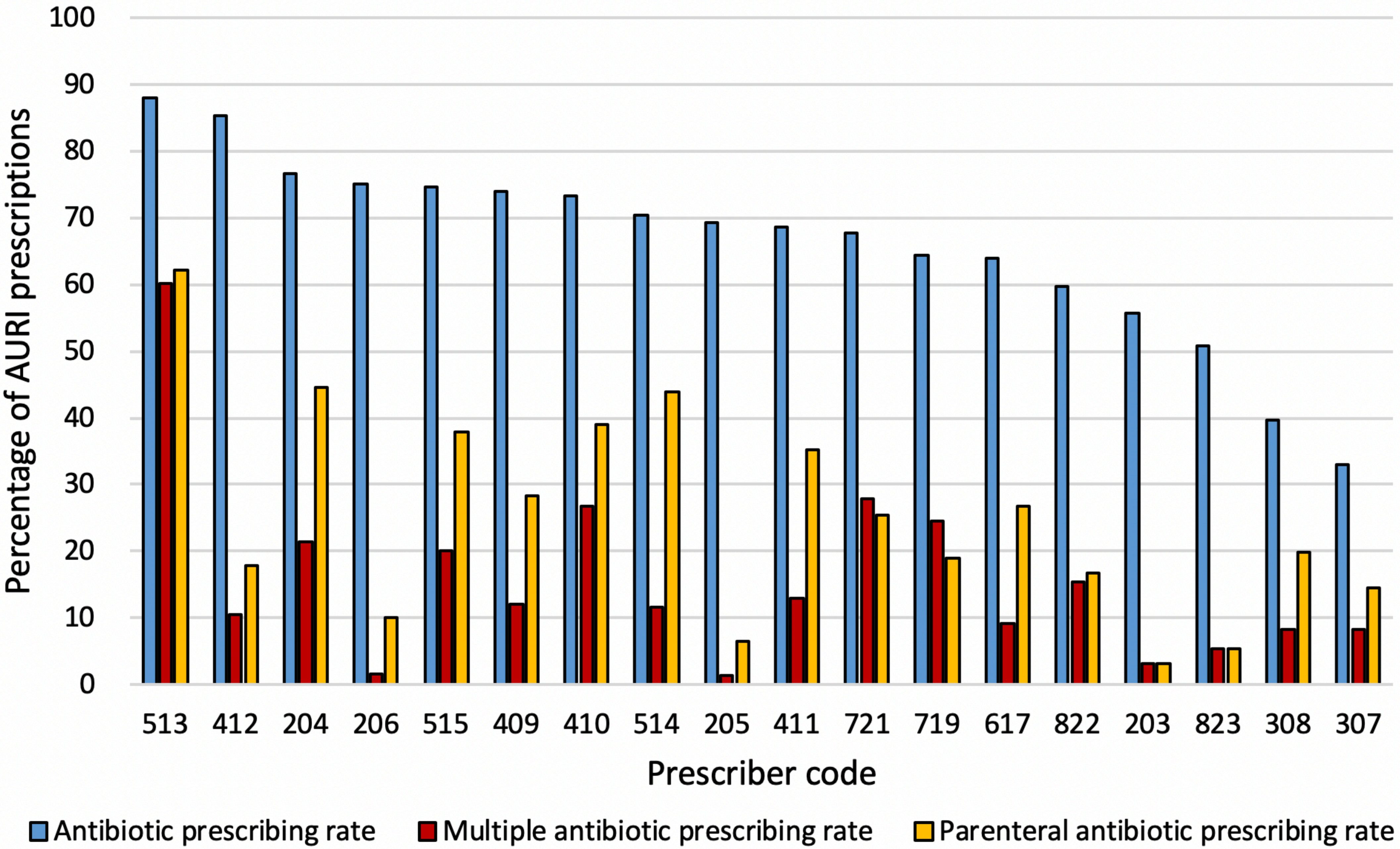
100
90
80
70
60
50
40
30
20
10
0
Percentage of AURI prescriptions
513
412
204
206
515
409
410
514
205
411
721
719
617
822
203
823
308
307
Prescriber code
Antibiotic prescribing rate
Multiple antibiotic prescribing rate
Parenteral antibiotic prescribing rate

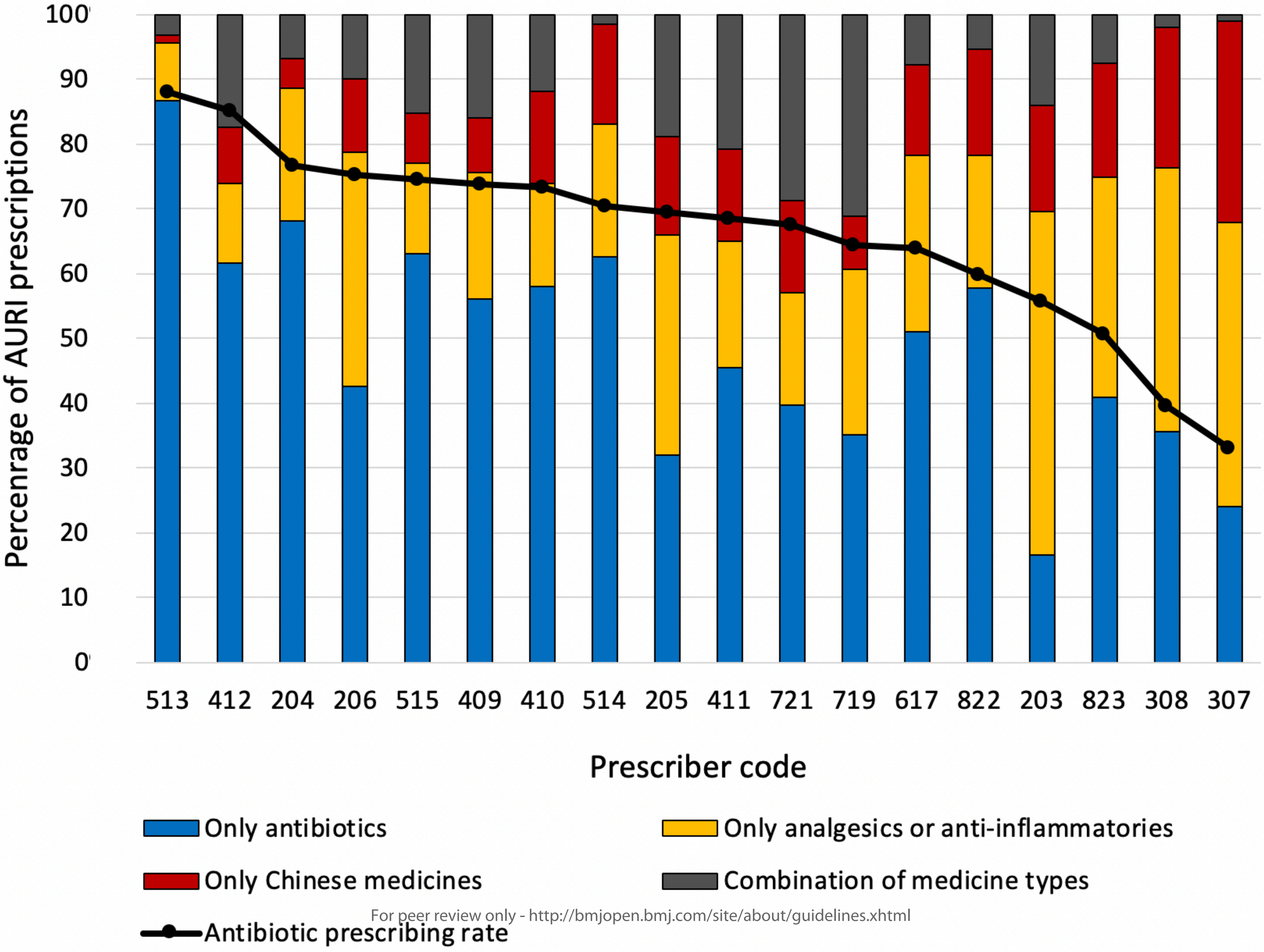
Percenrage of AURI prescriptions
100
90
80
70
60
50
40
30
20
10
0
513 412 204 206 515 409 410 514 205 411 721 719 617 822 203 823 308 307
Prescriber code
Only antibiotics
Only analgesics or anti-inflammatories
Only Chinese medicines
Combination of medicine types
Antibiotic prescribing rate

Appendix table 1. Class and agent of antibiotic prescribing for AURI (N = 3818)

Antibiotic class	Frequency	%	Antibiotic agent	Frequency	%
J01AA Tetracyclines	3	0.1	J01AA02 Doxycycline	3	0.1
J01CA Penicillins with Extended Spectrum	***1025***	***26.8***	J01CA01 Ampicillin	8	0.2
			J01CA04 Amoxicillin	***1017***	***26.6***
J01CE Beta-Lactamase Sensitive Penicillins	49	1.3	J01CE01 Benzylpenicillin	35	0.9
			J01CE02 Phenoxymethylpenicillin	14	0.4
J01CR Combinations of Penicillins, Incl. Beta-Lactamase Inhibitors	79	2.1	J01CR02 Amoxicillin And Enzyme Inhibitor	79	2.1
J01DB First-Generation Cephalosporins	***603***	***15.8***	***J01DB01 Cefalexin***	323	8.5
			J01DB09 Cefradine	280	7.3
J01DC Second-Generation Cephalosporins	272	7.1	J01DC02 Cefuroxime	272	7.1
J01DD Third-Generation Cephalosporins	255	6.7	J01DD01 Cefotaxime	193	5.1
			J01DD08 Cefixime	62	1.6
J01EE Combinations of Sulfonamides and Trimethoprim, Incl. Derivatives	1	0.0	J01EE01 Sulfamethoxazole	1	0.0
J01FA Macrolides	***516***	***13.5***	J01FA06 Roxithromycin	***317***	***8.3***
			J01FA09 Clarithromycin	7	0.2
			J01FA10 Azithromycin	192	5.0
J01FF Lincosamides	343	9.0	J01FF01 Clindamycin	45	1.2
			J01FF02 Lincomycin	298	7.8
J01GB Other Aminoglycosides	166	4.3	J01GB03 Gentamicin	37	1.0
			J01GB06 Amikacin	129	3.4
J01MA Fluoroquinolones	***423***	***11.1***	J01MA02 Ciprofloxacin	64	1.7
			J01MA06 Norfloxacin	25	0.7
			J01MA12 Levofloxacin	***334***	***8.7***
J01MB Other Quinolones	20	0.5	J01MB04 Pipemidic Acid	20	0.5
J01XD Imidazole Derivatives	58	1.5	J01XD01 Metronidazole	8	0.2
			J01XD02 Tinidazole	50	1.3
J01XE Nitrofuran Derivatives	3	0.1	J01XE01 Nitrofurantoin	3	0.1
J01XX Other Antibacterials	2	0.1	J01XX01 Fosfomycin	2	0.1

For peer review only

For peer review only - http://bmjopen.bmj.com/site/about/guidelines.xhtml

Appendix Table 2. Additional antibiotic prescribing rate indicators for individual prescribers

Prescriber	Clinic	Sex	Age	% of respiratory tract infection diagnoses coded as AURI			APR of AURI in 2015			APR of AURI in 2016			APR of potentially bacterial URTIs			APR of Gastroenteritis, diarrhoea and colitis		
				n	N	%	n	N	%	n	N	%	n	N	%	n	N	%
101	BFZG	2	46	377	647	58.3	151	181	83.4	70	142	49.3	56	61	91.8	58	62	93.5
102	BFZG	1	52	56	76	73.7	NA	NA	NA	NA	NA	NA	9	9	100.0	NA	NA	NA
203	CS	1	59	167	251	66.5	12	26	46.2	40	71	56.3	63	83	75.9	2	2	100.0
204	CS	1	46	73	126	57.9	17	22	77.3	21	28	75.0	50	52	96.2	10	10	100.0
205	CS	2	50	111	141	78.7	37	63	58.7	19	24	79.2	20	27	74.1	3	3	100.0
206	CS	1	63	173	216	80.1	57	87	65.5	73	86	84.9	32	39	82.1	10	10	100.0
307	DS	2	40	520	605	86.0	88	199	44.2	38	160	23.8	39	51	76.5	64	111	57.7
308	DS	1	67	217	251	86.5	57	132	43.2	29	84	34.5	20	28	71.4	33	70	47.1
409	FJZZ	1	63	134	136	98.5	54	77	70.1	45	57	78.9	NA	NA	NA	21	46	45.7
410	FJZZ	1	40	199	200	99.5	82	123	66.7	64	76	84.2	NA	NA	NA	25	48	52.1
411	FJZZ	1	43	124	146	84.9	NA	NA	NA	26	38	68.4	7	9	77.8	12	15	80.0
412	FJZZ	1	60	271	285	95.1	98	108	90.7	92	118	78.0	2	2	100.0	38	69	55.1
513	QD	1	46	117	124	94.4	76	89	85.4	27	28	96.4	NA	NA	NA	4	4	100.0
514	QD	1	52	578	597	96.8	113	123	91.9	168	222	75.7	2	2	100.0	31	35	88.6
515	QD	1	65	181	192	94.3	80	102	78.4	55	79	69.6	NA	NA	NA	16	20	80.0
616	XJG	1	63	19	33	57.6	4	14	28.6	1	5	20.0	6	9	66.7	2	3	66.7
617	XJG	1	39	415	546	76.0	76	117	65.0	98	163	60.1	22	31	71.0	69	100	69.0
618	XJG	1	37	12	13	92.3	NA	NA	NA	8	12	66.7	0	1	0.0	12	17	70.6
719	YJ	1	42	386	437	88.3	104	153	68.0	78	129	60.5	1	1	100.0	33	38	86.8
720	YJ	1	63	3	3	100.0	0	1	0.0	1	2	50.0	NA	NA	NA	NA	NA	NA
721	YJ	1	36	408	416	98.1	114	152	75.0	108	163	66.3	1	1	100.0	30	35	85.7
822	YJZZ	1	68	378	407	92.9	127	184	69.0	99	194	51.0	9	20	45.0	46	50	92.0
823	YJZZ	2	55	258	335	77.0	69	95	72.6	30	67	44.8	23	35	65.7	38	38	100.0

Footnote: APR = antibiotic prescribing rate; potentially bacterial upper respiratory tract infections (URTIs) = prescription containing and diagnosis of tracheitis, pharyngitis, tonsillitis or laryngopharyngitis. Rows in grey are not included in the individual prescriber-level analyses in the main text due to contributing <50 AURI prescriptions (prescribers 616, 618, 720) and due to an error in the coding of prescriber (prescribers 101, 102). 1 = male; 2 = female.

For peer review only - http://bmjopen.bmj.com/site/about/guidelines.xhtml

Appendix table 3. Most common antibiotic agents prescribed for AURI at individual prescriber level

Prescriber	Clinic	Sex	Age	1st most common antibiotic prescribed for AURI, name (no.)			2nd most common antibiotic prescribed for AURI, name (no.)			3rd most common antibiotic prescribed for AURI, name (no.)			Sum top 3	Total no. abx	% abx that are top three	No. abx agents total
101	BFZG	2	46	J01CA04	Amoxicillin	126	J01DB01	Cefalexin	35	J01FA06	Roxithromycin	31	192	247	77.7	13
102	BFZG	1	52	J01CA04	Amoxicillin	9							9	9	100.0	1
203	CS	1	59	J01MA12	Levofloxacin	25	J01DB09	Cefradine	21	J01CA04	Amoxicillin	16	62	93	66.7	13
204	CS	1	46	J01MA12	Levofloxacin	16	J01CA04	Amoxicillin	13	J01DD01	Cefotaxime	12	41	56	73.2	11
205	CS	2	50	J01CA04	Amoxicillin	21	J01DB09	Cefradine	12	J01MA12	Levofloxacin	12	45	77	58.4	12
206	CS	1	63	J01CA04	Amoxicillin	46	J01DB09	Cefradine	23	J01DB01	Cefalexin	19	88	130	67.7	11
307	DS	2	40	J01CA04	Amoxicillin	35	J01MA02	Ciprofloxacin	23	J01FA06	Roxithromycin	20	78	172	45.3	20
308	DS	1	67	J01CA04	Amoxicillin	24	J01DB01	Cefalexin	15	J01DB09<br>J01DC02<br>J01MA02	Cefradine<br>Cefuroxime<br>Ciprofloxacin	9<br>9<br>9	48<br>66	86	55.8<br>76.7	17
409	FJZZ	1	63	J01CA04	Amoxicillin	20	J01DB09	Cefradine	17	J01FA06	Roxithromycin	16	53	99	53.5	17
410	FJZZ	1	40	J01CA04	Amoxicillin	25	J01FA06	Roxithromycin	23	J01FF02	Lincomycin	23	71	146	48.6	18
411	FJZZ	1	43	J01FF02	Lincomycin	22	J01CA04	Amoxicillin	18	J01MA12	Levofloxacin	12	52	85	61.2	11
412	FJZZ	1	60	J01CA04	Amoxicillin	60	J01DB09	Cefradine	46	J01FA06	Roxithromycin	39	145	231	62.8	16
513	QD	1	46	J01GB06	Amikacin	33	J01DD01	Cefotaxime	28	J01MA12	Levofloxacin	28	89	103	86.4	16
514	QD	1	52	J01CA04	Amoxicillin	140	J01FF02	Lincomycin	137	J01DB09	Cefradine	28	305	407	74.9	21
515	QD	1	65	J01CA04	Amoxicillin	53	J01FF02	Lincomycin	27	J01GB06	Amikacin	15	95	135	70.4	18
616	XJG	1	63	J01CA04	Amoxicillin	1	J01CR02	Amoxicillin and Enzyme Inhibitor	1	J01DB01	Cefalexin	1	3	5	60.0	6
617	XJG	1	39	J01FA10	Azithromycin	49	J01CA04	Amoxicillin	46	J01FA06	Roxithromycin	42	137	265	51.7	19
618	XJG	1	37	J01CA04	Amoxicillin	2	J01DC02	Cefuroxime	2	J01DB09	Cefradine	1	5	8	62.5	7
719	YJ	1	42	J01CA04	Amoxicillin	86	J01DC02	Cefuroxime	43	J01DB01	Cefalexin	39	168	249	67.5	20
720	YJ	1	63	J01GB06	Amikacin	1	J01AA02						1	1	100.0	1

For peer review only

For peer review only - http://bmjopen.bmj.com/site/about/guidelines.xhtml

721	YJ	1	36	J01CA04	Amoxicillin	68	J01MA12	Levofloxacin	59	J01FA06	Roxithromycin	43	170	276	61.6	20
822	YJZZ	1	68	J01CA04	Amoxicillin	112	J01FA06	Roxithromycin	26	J01DC02	Cefuroxime	25	163	226	72.1	17
823	YJZZ	2	55	J01CA04	Amoxicillin	75	J01DB09	Cefradine	18	J01DB01	Cefalexin	14	107	131	81.7	13

Footnote: abx = antibiotics; 1 = male; 2 = female. Rows in grey are not included in the individual prescriber-level analyses in the main text due to contributing <50 AURI prescriptions (prescribers 616, 618, 720) and due to an error in the coding of prescriber (prescribers 101, 102).

For peer review only

Appendix table 4. Antibiotic use for AURI at level of individual prescriber

Prescriber	Clinic	Sex	Age	Patient age			Female patients		APR for ALL prescriptions			APR for AURI			MPR for AURI			PAPR for AURI			AURI with analgesic or anti-inflammatory		
				Mean	Min	Max	n	%	n	N	%	n	N	%	n	N	%	n	N	%	n	N	%
101	BFZG	2	46	48	2	90	152	40.3	794	1674	47.4	247	377	65.5	24	247	9.7	10	247	4.0	260	377	69.0
102	BFZG	1	52	50	13	78	19	33.9	37	185	20.0	9	56	16.1	0	9	0.0	0	9	0.0	35	56	62.5
203	CS	1	59	53	5	81	53	31.7	226	598	37.8	93	167	55.7	3	93	3.2	3	93	3.2	127	167	76.0
204	CS	1	46	56	36	75	37	50.7	165	283	58.3	56	73	76.7	12	56	21.4	25	56	44.6	34	73	46.6
205	CS	2	50	52	9	80	50	45.0	142	357	39.8	77	111	69.4	1	77	1.3	5	77	6.5	66	111	59.5
206	CS	1	63	49	5	86	62	35.8	246	560	43.9	130	173	75.1	2	130	1.5	13	130	10.0	109	173	63.0
307	DS	2	40	50	1	90	206	39.6	372	1255	29.6	172	520	33.1	14	172	8.1	25	172	14.5	229	520	44.0
308	DS	1	67	47	4	89	95	43.8	171	576	29.7	86	217	39.6	7	86	8.1	17	86	19.8	78	217	35.9
409	FJZZ	1	63	54	5	84	64	47.8	138	328	42.1	99	134	73.9	12	99	12.1	28	99	28.3	55	134	41.0
410	FJZZ	1	40	53	3	86	95	47.7	191	405	47.2	146	199	73.4	39	146	26.7	57	146	39.0	80	199	40.2
411	FJZZ	1	43	56	10	83	57	46.0	144	365	39.5	85	124	68.5	11	85	12.9	30	85	35.3	61	124	49.2
412	FJZZ	1	60	54	5	90	137	50.6	329	654	50.3	231	271	85.2	24	231	10.4	41	231	17.7	134	271	49.4
513	QD	1	46	47	10	85	57	48.7	138	197	70.1	103	117	88.0	62	103	60.2	64	103	62.1	20	117	17.1
514	QD	1	52	49	2	92	286	49.5	528	1111	47.5	407	578	70.4	47	407	11.5	179	407	44.0	205	578	35.5
515	QD	1	65	49	3	83	86	47.5	196	417	47.0	135	181	74.6	27	135	20.0	51	135	37.8	83	181	45.9
616	XJG	1	63	56	5	82	4	21.1	34	101	33.7	5	19	26.3	1	5	20.0	2	5	40.0	7	19	36.8
617	XJG	1	39	44	1	87	156	37.6	536	1634	32.8	265	415	63.9	24	265	9.1	71	265	26.8	173	415	41.7
618	XJG	1	37	44	10	63	4	33.3	27	91	29.7	8	12	66.7	1	8	12.5	1	8	12.5	6	12	50.0
719	YJ	1	42	54	2	90	178	46.1	416	923	45.1	249	386	64.5	61	249	24.5	47	249	18.9	225	386	58.3
720	YJ	1	63	60	34	73	3	100.0	1	5	20.0	1	3	33.3	0	1	0.0	1	1	100.0	2	3	66.7
721	YJ	1	36	57	4	91	150	36.8	415	928	44.7	276	408	67.6	77	276	27.9	70	276	25.4	202	408	49.5
822	YJZZ	1	68	50	2	87	171	45.2	338	1038	32.6	226	378	59.8	35	226	15.5	38	226	16.8	159	378	42.1
823	YJZZ	2	55	48	2	86	119	46.1	249	786	31.7	131	258	50.8	7	131	5.3	7	131	5.3	141	258	54.7

Footnote: APR = antibiotic prescribing rate; MPR = multiple antibiotic prescribing rate; PAPR = parenteral antibiotic prescribing rate; 1 = male; 2 = female. Rows in grey are not included in the individual prescriber-level analyses in the main text due to contributing <50 AURI prescriptions (prescribers 616, 618, 720) and due to an error in the coding of prescriber

For peer review only - http://bmjopen.bmj.com/site/about/guidelines.xhtml

(prescribers 101, 102).

Appendix Table 5. Different types of medicines used by individual prescribers for AURI

Prescriber	Clinic	Sex	Age	AURI rx	Only abx		Only inf		Only TCM		Abx plus inf		Abx plus TCM		Inf plus TCM		Abx plus inf plus TCM		Only other drugs		Ratio only abx:only inf
				n	n	%	n	%	n	%	n	%	n	%	n	%	n	%	n	%	
101	BFZG	2	46	377	19	5.0	26	6.9	29	7.7	70	18.6	65	17.2	71	18.8	93	24.7	4	1.1	0.7
102	BFZG	1	52	56	0	0.0	30	53.6	17	30.4	5	8.9	4	7.1	0	0	0	0	0	0	0
203	CS	1	59	167	13	7.8	42	25.1	13	7.8	61	36.5	8	4.8	13	7.8	11	6.6	6	3.6	0.3
204	CS	1	46	73	30	41.1	9	12.3	2	2.7	19	26	4	5.5	3	4.1	3	4.1	3	4.1	3.3
205	CS	2	50	111	17	15.3	18	16.2	8	7.2	33	29.7	17	15.3	5	4.5	10	9	3	2.7	0.9
206	CS	1	63	173	34	19.7	29	16.8	9	5.2	69	39.9	19	11	3	1.7	8	4.6	2	1.2	1.2
307	DS	2	40	520	92	17.7	167	32.1	118	22.7	41	7.9	35	6.7	17	3.3	4	0.8	46	8.8	0.6
308	DS	1	67	217	54	24.9	62	28.6	33	15.2	10	4.6	19	8.8	3	1.4	3	1.4	33	15.2	0.9
409	FJZZ	1	63	134	46	34.3	16	11.9	7	5.2	22	16.4	18	13.4	4	3	13	9.7	8	6	2.9
410	FJZZ	1	40	199	73	36.7	20	10.1	18	9.0	36	18.1	22	11.1	9	4.5	15	7.5	6	3	3.7
411	FJZZ	1	43	124	35	28.2	15	12.1	11	8.9	19	15.3	15	12.1	11	8.9	16	12.9	2	1.6	2.3
412	FJZZ	1	60	271	92	33.9	18	6.6	13	4.8	82	30.3	31	11.4	8	3	26	9.6	1	0.4	5.1
513	QD	1	46	117	79	67.5	8	6.8	1	0.9	9	7.7	12	10.3	0	0	3	2.6	5	4.3	9.9
514	QD	1	52	578	264	45.7	86	14.9	65	11.2	100	17.3	37	6.4	13	2.2	6	1	7	1.2	3.1
515	QD	1	65	181	58	32.0	13	7.2	7	3.9	40	22.1	23	12.7	16	8.8	14	7.7	10	5.5	4.5
616	XJG	1	63	19	3	15.8	5	26.3	6	31.6	1	5.3	1	5.3	1	5.3	0	0	2	10.5	0.6
617	XJG	1	39	415	156	37.6	83	20.0	42	10.1	55	13.3	30	7.2	11	2.7	24	5.8	14	3.4	1.9
618	XJG	1	37	12	4	33.3	3	25.0	0	0.0	3	25	1	8.3	0	0	0	0	1	8.3	1.3
719	YJ	1	42	386	83	21.5	61	15.8	19	4.9	42	10.9	50	13	48	12.4	74	19.2	9	2.3	1.4
720	YJ	1	63	3	1	33.3	1	33.3	0	0.0	0	0	0	0	1	33.3	0	0	0	0	1
721	YJ	1	36	408	94	23.0	41	10.0	34	8.3	62	15.2	52	12.7	31	7.6	68	16.7	26	6.4	2.3
822	YJZZ	1	68	378	128	33.9	46	12.2	36	9.5	52	13.8	34	9	49	13	12	3.2	21	5.6	2.8
823	YJZZ	2	55	258	59	22.9	49	19.0	25	9.7	43	16.7	18	7	38	14.7	11	4.3	15	5.8	1.2

For peer review only - http://bmjopen.bmj.com/site/about/guidelines.xhtml

Footnote: rx = prescriptions; abx = antibiotic; inf = analgesic or anti-inflammatory medicines (any of the following: aspirin/acetylsalicylic acid, ibuprofen, paracetamol or diclofenac); TCM = traditional Chinese medicine; 1 = male; 2 = female. Rows in grey are not included in the individual prescriber-level analyses in the main text due to contributing <50 AURI prescriptions (prescribers 616, 618, 720) and due to an error in the coding of prescriber (prescribers 101, 102).

For peer review only

BMJ Open

Variations in antibiotic prescribing among village doctors in a rural region of Shandong province, China: a cross-sectional analysis of prescriptions

Journal:	*BMJ Open*
Manuscript ID	bmjopen-2019-036703.R1
Article Type:	Original research
Date Submitted by the Author:	04-Mar-2020
Complete List of Authors:	Dyar, Oliver; Karolinska Institutet, Department of Global Public Health<br>Ding, Yang; Shandong University, School of Health Care Management<br>Yin, Jia; Shandong University, School of Health Care Management<br>Sun, Qiang; Shandong University, School of Health Care Management<br>Lundborg, Cecilia; Karolinska Institutet, Department of Global Public Health
**Primary Subject Heading**:	Public health
Secondary Subject Heading:	General practice / Family practice, Infectious diseases
Keywords:	PRIMARY CARE, Public health < INFECTIOUS DISEASES, Quality in health care < HEALTH SERVICES ADMINISTRATION & MANAGEMENT, Respiratory infections < THORACIC MEDICINE

SCHOLARONE™
Manuscripts

I, the Submitting Author has the right to grant and does grant on behalf of all authors of the Work (as defined in the below author licence), an exclusive licence and/or a non-exclusive licence for contributions from authors who are: i) UK Crown employees; ii) where BMJ has agreed a CC-BY licence shall apply, and/or iii) in accordance with the terms applicable for US Federal Government officers or employees acting as part of their official duties; on a worldwide, perpetual, irrevocable, royalty-free basis to BMJ Publishing Group Ltd ("BMJ") its licensees and where the relevant Journal is co-owned by BMJ to the co-owners of the Journal, to publish the Work in this journal and any other BMJ products and to exploit all rights, as set out in our licence.

The Submitting Author accepts and understands that any supply made under these terms is made by BMJ to the Submitting Author unless you are acting as an employee on behalf of your employer or a postgraduate student of an affiliated institution which is paying any applicable article publishing charge ("APC") for Open Access articles. Where the Submitting Author wishes to make the Work available on an Open Access basis (and intends to pay the relevant APC), the terms of reuse of such Open Access shall be governed by a Creative Commons licence – details of these licences and which Creative Commons licence will apply to this Work are set out in our licence referred to above.

Other than as permitted in any relevant BMJ Author's Self Archiving Policies, I confirm this Work has not been accepted for publication elsewhere, is not being considered for publication elsewhere and does not duplicate material already published. I confirm all authors consent to publication of this Work and authorise the granting of this licence.

For peer review only

Title: Variations in antibiotic prescribing among village doctors in a rural region of Shandong province, China: a cross-sectional analysis of prescriptions

Oliver J. Dyar[a]*, Yang Ding[b]*, Jia Yin[b], Qiang Sun[b+], Cecilia Stålsby Lundborg[a]

[a]Department of Global Public Health, Karolinska Institutet, Stockholm, Sweden

[b]School of Health Care Management, Shandong University, Jinan, Shandong, China; Key Laboratory of Health Economics and Policy Research, NHC, Shandong University, Jinan, Shandong, China; Center for Health Management and Policy Research, Shandong University, Jinan, Shandong, China

*Contributed equally

[+]Corresponding author: School of Health Care Management, Shandong University, Jinan, Shandong, China. Tel: +86-531-88382376; Fax: +86-531-88382693; E-mail: qiangs@sdu.edu.cn.

For peer review only

Abstract

Objectives: To assess variation in antibiotic prescribing practices among village doctors in a rural region of Shandong province, China.

Design, setting and participants: Almost all outpatient encounters at village clinics result in a prescription being issued. Prescriptions were collected over a 2.5 year period from eight primary care village clinics staffed by 23 doctors located around a town in rural Shandong province. A target of 60 prescriptions per clinic per month were sampled from an average total of around 300. Prescriptions were analysed at both aggregate and individual-prescriber levels, with a focus on diagnoses of likely viral acute upper respiratory tract infections (AURI), defined as ICD-10 codes J00 and J06.9.

Main outcome measures: Proportions of prescriptions for AURIs containing i) at least one antibiotic, ii) multiple antibiotics, iii) at least one parenteral antibiotic; classes and agents of antibiotics prescribed.

Results: In total 14471 prescriptions from 23 prescribers were included, of which 5833 (40.3%) contained at least one antibiotic. Nearly two-thirds 62.5% (3237/5177) of likely viral AURI prescriptions contained an antibiotic, accounting for 55.5% (3237/5833) of all antibiotic-containing prescriptions. There was wide variation at the individual-level in antibiotic prescribing rates for AURIs (33.1% to 88.0%), as well multiple antibiotic prescribing rates (1.3% to 60.2%) and parenteral antibiotic prescribing rates (3.2% to 62.1%). Each doctor prescribed between 11 and 21 unique agents for AURIs, including many broad-spectrum antibiotics. Doctors in the highest quartile for antibiotic prescribing rates for AURI also had higher antibiotic prescribing rates than doctors in the lowest quartile for potentially bacterial upper respiratory tract infections (pharyngitis, tonsillitis, laryngopharyngitis) (89.1% vs. 72.4%, p=0.002).

Conclusions: All village doctors over-used antibiotics for respiratory tract infections. Variations in individual prescriber practices are significant even in a small homogenous setting and should be accounted for when developing targets and interventions to improve antibiotic use.

KEYWORDS

Primary care; Public health; Quality in health care; Respiratory infections

ARTICLE SUMMARY

Strengths and limitations of this study

- We investigated whether significant variations exist in antibiotic prescribing patterns among village doctors working in a small, homogenous setting in rural China
- We included over 14000 prescriptions sampled across a 2.5 year time period
- Uniquely, we analysed prescriptions according to clinical diagnosis, focussing on acute upper respiratory tract infections, and at the level of individual prescribers
- We could not verify diagnoses clinically, and so variations between the prescribers in terms of diagnostic ability could not be assessed

For peer review only

BACKGROUND

Irrational antibiotic use is an important and modifiable driver of antibiotic resistance.[1] A knowledge of the specific patterns of unnecessary or inappropriate antibiotic use is important for designing context-adapted, targeted interventions to improve antibiotic use, such as education focussing on common irrational practices within a region or institution.[2] Understanding the variations that occur in antibiotic prescribing patterns at the prescriber-level can further be valuable for shedding light on the facilitators and barriers to rational antibiotic use, as well as for enabling individualised feedback on prescribing practices.[3,4]

Previous studies in China strongly indicate that there is widespread antibiotic overuse in outpatient clinics in rural areas, particularly for respiratory tract infections.[5–8] These studies have not been capable of investigating the extent of variability in practices that exist, due to two common limitations: first, most studies have relied on highly aggregated data (e.g. at the level of an institution or county); second, some studies have not been able to couple antibiotic prescriptions with clinical diagnoses at the level of the individual prescription. Recent studies in other countries have highlighted the value of such finer-grained analyses; for example, Pouwels *et al.* found that variability in prescribing patterns for antibiotics at primary care centres in the UK could not be adequately explained by variations in patient consultation rate or pre-existing co-morbidities,[9] i.e. individual prescriber behaviours are an important cause of irrational antibiotic use, and so need to be addressed.[10]

We investigated patterns of antibiotic prescriptions at the individual prescriber-level in eight village clinics in rural Shandong province, China, over a two-and-a-half-year time period. Village clinics are the first place that most rural residents seek healthcare, and almost all consultations result in patients receiving a prescription, in part because prescriptions are a way in which reimbursements for healthcare expenditures are calculated and administered. The clinics are staffed by village doctors who typically have a high school level education and additional vocation-related training; many of the older village doctors were trained under the barefoot doctor programme.[11] Our aims were to assess if there was overall evidence of irrational antibiotic use, and whether there was significant variability between the practices of individual prescribers in this small, homogenous region. Our study forms part of a large cross-sectoral research programme on antibiotic consumption and resistance involving Chinese and Swedish researchers, the Sino-Swedish Integrated Multi-sectoral Partnership for Antibiotic Resistance Containment (IMPACT).[12]

MATERIALS AND METHODS

Study design and setting

We conducted a prospective observational analysis of outpatient prescriptions from January 2015 to July 2017 at eight village clinics clustered around a single town in Z County, Shandong province. This county is in the middle level in Shandong province in terms of economics, health indicators and population size. As previously described in the full study protocol,[12] the county and town were first selected based on the requirements and design of the IMPACT research programme, in particular being broadly representative of rural Shandong province, and including the presence of local collaborators who were capable of providing administrative support throughout the entire duration of the research programme. Twelve villages were then purposively selected around the town from among seventeen that had at least one hundred households. These twelve villages are

For peer review only

served by eight village clinics located in the villages and are attended by 23 doctors who do not move between clinics.

Data collection

The prescriptions of all village clinics in Z county are included in an e-prescription system that records for each visit the patients' name, sex, age, date of visit, diagnosis, medicines prescribed, total cost for the visit and name of the doctor. For each village clinic a target of 60 prescriptions per month (from an average total of around 300) were collected using a random sampling method in which every fifth consecutive prescription was selected, beginning with a randomly generated starting number between 1 and 10. The prescription details were exported one-by-one from the e-prescription system in XPS format and entered into a Microsoft Excel database. In addition, in July 2015 the doctors completed a short face-to-face questionnaire concerning their demographic information (gender, age, number of years working).

Data management and analyses

Prescriptions from one doctor who did not complete the questionnaire were removed (55 in total). Data were then anonymised with patient names being removed and each doctor assigned a three-digit code representing the village clinic at which they worked (1XX to 8XX) and a unique number (X01 to X23).

All diagnoses on prescriptions were coded where possible according to the International Classification of Diseases, 10th Revision (ICD-10) by an independent researcher from China with a background in clinical medicine and public health: first, a list of unique diagnoses included on the prescriptions was created; second, these were matched with the standard ICD-10 Chinese translation provided by the National Health Commission of China that is used in hospitals; finally, these diagnoses were translated into English. In cases of uncertainty a shortlist of potential diagnoses was discussed between the first author (a clinical doctor and public health researcher from Europe) and the independent researcher until either agreement was reached or no ICD-10 code was assigned (e.g. when a symptom was listed instead of a diagnosis, or when it was unclear whether a diagnosis was acute or chronic); in total, these cases of uncertainty accounted for <10% of all prescriptions, and did not include any prescriptions that were ultimately classified as likely viral acute upper respiratory tract infections. A category of *likely viral acute upper respiratory tract infections* (AURI) was created by grouping diagnoses of J00 (acute nasopharyngitis [common cold]) and J06.9 (acute upper respiratory infection, unspecified).

Prescriber-level analyses were limited to the 20/23 doctors with ≥50 AURI prescriptions during the study period, to ensure that the antibiotic prescribing rates calculated for each individual doctor were sufficiently representative of their practice. Of the three doctors with fewer than 50 AURI prescriptions, two retired from clinical practice near the beginning of the study period (616 and 720), and one newly started working at the specific village clinic at the end of the study period (618). We identified an error in the coding of prescriptions in village clinic 1 which was due to one doctor (101) being responsible for entering the majority of prescriptions onto the electronic system using their account, irrespective of who the actual prescriber was, because of the low computer literacy of the other doctor (102). We therefore could not be confident of which doctor was responsible for which prescription, so all 433 prescriptions from the two doctors working at this clinic were excluded from prescriber-level analyses. This error was not present at other village

For peer review only

clinics. All prescribed antibiotics were coded using the Anatomical Therapeutic Chemical (ATC) classification system[13] and categorised by classes and substances. Prescribed medicines were also coded as: "Analgesic or anti-inflammatory medicines" (aspirin/acetylsalicylic acid, ibuprofen, paracetamol or diclofenac); "traditional Chinese medicines"; or "other".

For each prescriber, antibiotic prescribing was analysed using three indicators: a) antibiotic prescribing rate (APR) = number of prescriptions that included at least one antibiotic / total number of prescriptions x 100%, b) multiple antibiotics prescribing rate (MPR) = number of prescriptions that included at least two antibiotics with different ATC codes / total number of prescriptions that included at least one antibiotic x 100%, c) parenteral antibiotic prescribing rate (PAPR) = number of prescriptions that included at least one parenteral antibiotic / total number of prescriptions that included at least one antibiotic x 100%. Comparisons were also made against antibiotic prescribing quality indicators developed in Europe[14] for a subset of clinical diagnoses and patient ages.

All analyses were conducted in Microsoft Excel and SPSS. Descriptive statistics were calculated and comparisons were made using χ^2 tests and Pearson correlation. The Kruskal-Wallis H-test was used to assess whether there were statistically significant variations between the individual doctors in terms of antibiotic prescribing rates for *likely viral AURIs*. Pairwise Mann-Whitney U-tests were used post-hoc to assess for differences in all pairwise comparisons between doctors, using the Benjamini-Hochberg procedure [15] to control the false discovery rate for multiple comparisons. Statistical significance was set at $p < 0.05$ for all tests. We used the Strengthening the Reporting of Observational Studies in Epidemiology guidelines for reporting the results of cross-sectional observational study (see online supplementary file).

Patient and public involvement

Patients or the public were not involved in the design, conduct, reporting, or dissemination plans of this specific study, but have been involved in the dissemination plans for results of the overall IMPACT research programme.

Ethics

Informed consent was obtained from all village doctors. Ethical approval was obtained from the First Affiliated Hospital, College of Medicine, Zhejiang University, China (reference numbers 2015#185 and 2015#283).

RESULTS

Overview of prescriptions and diagnoses

In total 14471 prescriptions from 23 prescribers from January 2015 to July 2017 were included in the analyses. This included an average of 58 prescriptions per month per village clinic (mean range from 47 to 62). There was a total of 222 diagnoses in Chinese, with 20 diagnoses accounted for nearly 90% of all prescriptions (12811/14471). The five most common diagnoses overall were: J00 (acute nasopharyngitis [common cold], 34.1%, 4938/14471), I10 (Essential hypertension, 13.6%, 1966/14471), K29.7 (Gastritis, unspecified, 5.9%, 856/14471), M13.9 (Arthritis, unspecified, 5.9%, 955/14471) and I25.1 (Atherosclerotic heart disease, 3.2%, 470/14471). Overall, 40.3% of the prescriptions (5833/14471) contained at least one antibiotic.

For peer review only

Table 1 summarises the infection-related diagnoses that had a minimum of ten prescriptions during the study period. Overall, respiratory tract infections accounted for 68.4% (3990/5833) and gastrointestinal conditions for 14.0% (815/5833) of all prescriptions containing at least one antibiotic. A total of 5177 prescriptions were categorised as *likely viral acute upper respiratory tract infections* (AURI), and 62.5% (3237/5177) of these prescriptions contained at least one antibiotic, accounting for 55.5% (3237/5833) of all antibiotic-containing prescriptions. Fifteen percent (491/3237) of AURI antibiotic prescriptions included two or more antibiotics and 24.3% (785/3237) included at least one parenteral antibiotic.

Table 1. Infection-related diagnoses and antibiotic prescription rates

Body system (No. of prescriptions)	Diagnosis	ICD-10 code	Antibiotic prescribing rate n/N	%
Respiratory (6178)	Acute nasopharyngitis [common cold]+	J00	3034/4938	61.4
	Bronchitis*	J40	408/522	78.2
	Acute upper respiratory infection+	J06.9	203/239	84.9
	Acute pharyngitis	J02.9	160/190	84.2
	Acute tonsillitis	J03.9	68/73	93.2
	Rhinitis*	NA	31/67	46.3
	Cough	NA	23/65	35.4
	Pneumonia	J18	40/51	78.4
	Bronchopneumonia	J18.0	15/19	78.9
	Acute laryngopharyngitis	J06.0	8/14	57.1
Gastrointestinal (1642)	Gastritis	K29.7	258/856	30.1
	Gastroenteritis	A09.9	496/717	69.2
	Diarrhoea	NA	61/69	88.4
Dental (222)	Chronic periodontitis	K05.3	100/131	76.3
	Gingivitis and periodontal diseases	K05	61/72	84.7
	Pulpitis	K04.0	19/19	100.0
Urogenital (84)	Inflammatory disease of prostate	N41.9	23/45	51.1
	Urethritis and urethral syndrome	N34	23/24	95.8
	Urinary tract infection	N39.0	15/15	100.0
Eye (101)	Conjunctivitis	H10	9/101	8.9
Ear (11)	Otitis media	H66.9	7/11	63.6

Table footnote: *Not specified as acute or chronic; +Categorised as likely viral acute upper respiratory tract infection (AURI); APR = antibiotic prescription rate; NA = no ICD-10 code was allocated.

Medicines prescribed for AURI

Figure 1 shows the combinations of different medicine types (antibiotics, anti-inflammatories or analgesics, and traditional Chinese medicines) prescribed for AURI. Approximately half of all prescriptions with AURI (54.0%, 2795/5177) contained only one medicine type, a third (33.7%, 1744/5177) contained two medicine types, and 8% (414/5177) contained all three medicine types.

For peer review only

[Insert Figure 1]

Sixteen classes of antibiotics and 27 antibiotic agents were prescribed for AURI (Appendix Table 1). The three most common classes of antibiotics prescribed were: J01CA (Penicillins with extended spectrum, 31.6% of prescriptions), J01DB (First-generation cephalosporins, 18.6% of prescriptions), J01FA (Macrolides, 15.7% of prescriptions). The three most common agents of antibiotics prescribed for AURI were J01CA04 (Amoxicillin, 31.4% of prescriptions), J01MA12 (Levofloxacin, 10.3% of prescriptions), J01DB01 (Cefalexin, 9.9% of prescriptions).

Comparison with European quality indicators

Table 2 shows antibiotic prescribing rates and types of antibiotic for a variety of respiratory tract infections in our database based on previously published European recommendations.[14]

Table 2. **Antibiotic prescribing quality indicators for selected diagnoses**

Diagnosis	Patient age group (years)	APR n/N, %	Class of antibiotics		
			J01M n/N, %	J01CE n/N, %	J01AA or J01CA n/N, %
AURI	≥2	3236/5172, 62.6	436/3236, 13.5	49/3236, 1.5	
Target		*0-20%*	*0-5%*	*80-100%*	NA
Tonsillitis	≥2	68/73, 93.2	15/68, 22.1	2/68, 2.9	
Target		*0-20%*	*0-5%*	*80-100%*	NA
Pneumonia	18 to 65	22/30, 73.3	11/22, 50.0		2/22, 12.8
Target		*90-100%*	*0-5%*	NA	*80-100%*

Table footnote: APR = antibiotic prescribing rate; targets are as previously published,[14] except for "NA" which indicates that no target was included; J01M is the ATC code for Quinolone antibacterials, J01CE is the ATC code for Beta-lactamase sensitive penicillins, J01AA is the ATC code for Tetracyclines, J01CA is the ATC code for Penicillins with extended spectrum.

Prescriber demographics

Table 3 summarises prescribers' demographic information and antibiotic prescribing rates for patients with AURI. The median age of the prescribers was 51 years old, and the median years of working experience was 31. Male prescribers were more likely to prescribe antibiotics for their patients than female prescribers (67.7% versus 42.7%, $p < 0.01$). Male prescribers were slightly more lightly to prescribe antibiotics for female patients than for male patients (69.5% versus 66.3%, $p = 0.04$), but no difference was seen among female prescribers (41.9% versus 43.4%, $p = 0.65$).

Table 3. Prescriber demographics and antibiotic prescribing rates for AURIs

Prescriber	n	%	AURI prescriptions			
			n	APR (%)	MPR (%)	PAPR (%)
Total			*4710*	*63.0*	*15.7*	*26.0*
Gender						
Male	15	83.3	3821	67.7	17.1	28.4
Female	3	16.7	889	42.7	5.8	9.7
Age						
36~39	2	11.1	823	65.7	18.7	26.1
40~49	6	33.3	1419	57.2	24.5	30.6
50~59	4	22.2	1114	63.6	8.2	27.4
60~68	6	33.3	1354	67.0	11.8	20.7
Education*						
Junior high school	3	16.7	962	60.9	9.7	34.0
Senior high school	15	83.3	3748	63.5	17.1	24.0
Working years						
14~19	5	27.8	1666	56.7	17.5	26.8
20~29	4	22.2	687	70.6	28.0	29.1
30~39	2	11.1	836	64.4	10.0	34.6
40~48	7	38.9	1521	65.7	11.0	19.1

Table footnote: AURI = likely viral acute upper respiratory tract infections; APR = antibiotic prescribing rate; MPR = multiple antibiotics prescribing rate; PAPR = parenteral antibiotics prescribing rate. *Education: Junior high school only is equivalent to completing 9 years of full-time education; senior high school is equivalent to completing 12 years of full-time education.

For peer review only

Variations at the prescriber-level

Overall, individual prescribers' AURI antibiotic prescribing rates were highly correlated between 2015 and 2016 (r = 0.65). The mean absolute individual variation in AURI antibiotic prescribing rates between 2015 and 2016 was +/-13.1% (standard deviation = 6.7%). Eleven prescribers had lower AURI antibiotic prescribing rates in 2016 compared with 2015, and six had higher antibiotic prescribing rates in 2016 (Appendix Table 2).

Amoxicillin was the most commonly prescribed agent for AURI for 13/18 prescribers; the other agents that were the single most commonly prescribed for individual prescribers included levofloxacin (2 doctors), amikacin (1), lincomycin (1) and azithromycin (1). For each prescriber, their three most commonly prescribed antibiotic agents accounted for a mean of 64.4% (range 45.3% to 86.4%) of all the antibiotics they prescribed for AURI (Appendix Table 3).

Figure 2 and Appendix Table 4 illustrate the individual variations between the eighteen doctors for AURI prescriptions. There was wide variation in the antibiotic prescribing rate (33.1% to 88.0%), multiple antibiotic prescribing rate (1.3% to 60.2%) and parenteral antibiotic prescribing rate (3.2% to 62.1%). Village doctors' APRs were positively correlated with their MPRs (r = 0.47, p = 0.05) and PAPRs (r = 0.54, p = 0.02), but there was no association between doctors' APRs and the prescription of at least one analgesic or anti-inflammatory medicine (r = -0.19, p = 0.46). Village doctors' MPRs were also positively correlated with their PAPRs (r = 0.77, $p < 0.01$).

The Kruskal-Wallis H-test showed that the variations in antibiotic prescribing rates for AURI prescriptions were statistically significant among the 18 prescribers ($p < 0.001$). Pairwise Mann－Whitney U-tests were used as post-hoc tests to assess for differences in all possible pairwise comparisons between prescribers. This involved 153 pairs of prescribers, and the difference was statistically significant in 93 of these.

[Insert Figure 2]

Figure 3 and Appendix Table 5 illustrate the different types of medicines used for AURI prescriptions by 18 prescribers. The most common choice for prescribers (14/18) was to use only an antibiotic; the most common choice for the four remaining prescribers was to use only analgesic or anti-inflammatory medicines. All prescribers prescribed all three types of medicines for patients with a diagnosis of AURI on at least one occasion.

[Insert Figure 3]

Comparisons between high and low antibiotic prescribers

Table 4 shows comparisons for selected indicators between the four prescribers with the highest antibiotic prescribing rate for AURI (513, 412, 204 and 206) and the four prescribers with the lowest APR for AURI (203, 823, 308, 307).

Table 4. Variation between high and low AURI antibiotic prescribing rate groups

Indicator	High AURI APR group (%)	Low AURI APR group (%)	χ^2 *value*	*P value*
Mean APR for likely viral AURIs	81.3	44.8	273.31	<0.001
Mean MPR for likely viral AURIs	23.4	6.2	36.06	<0.001
Mean PAPR for likely viral AURIs	33.6	10.7	44.57	<0.001
Mean prescribing rate of only antibiotics for likely viral AURIs	40.6	18.3	72.88	<0.001
Mean prescribing rate of only analgesics or anti-inflammatory medicines for likely viral AURIs	10.6	26.2	74.26	<0.001
Mean APR for URTI with potential bacterial causes*#	89.1	72.4	9.25	0.002
Mean APR for gastritis, gastroenteritis and diarrhoea	85.0	68.3	9.17	0.002

Table footnote: The high AURI APR group contained the four village doctors with the highest antibiotic prescribing rates for likely viral AURIs, and the low AURI APR group contained the four village doctors with the lowest antibiotic prescribing rates for likely viral AURIs; APR = Antibiotic prescribing rate; MPR = Multiple antibiotics prescribing rate; PAPR = Parenteral antibiotics prescribing rate; *URTI with potential bacterial causes = prescriptions with an upper respiratory tract infection diagnosis of pharyngitis, tonsillitis or laryngopharyngitis; #These comparisons were restricted to village doctors in each group who had at least ten prescriptions containing a relevant diagnosis.

For peer review only

DISCUSSION

We prospectively investigated patterns of antibiotic prescriptions over a two-and-a-half-year period in village clinics in rural Shandong province in order to assess evidence of irrational antibiotic use and variability in prescribing practices at the level of the individual prescriber.

Consultation patterns and overall antibiotic use

The patterns of diagnoses on the prescriptions in our study are broadly similar to those in previous studies conducted in rural China,[5,16] with respiratory tract infections accounting for a large proportion of patient visits. We found that urogenital infections and skin and soft tissue infections each accounted for less than 1% of all antibiotic prescriptions at the village clinics, whereas these conditions are typically the second and third most common reasons for antibiotic prescriptions in studies in primary care clinics from high income countries [17]. In contrast, dental conditions were the third largest group of diagnoses for which antibiotics were prescribed in our study. This may reflect the extreme paucity of dentists in rural China [11], and is potentially an important and under-reported source of antibiotic overuse. Overall, forty percent of prescriptions contained at least one antibiotic. This antibiotic prescription rate is considerably higher than the government's target from 2012 of fewer than 20% outpatient prescriptions (for secondary level hospitals),[18,19] but is lower than previously published studies in China which tend to be around 50%.[5,7,16,20,21]

Antibiotic use for respiratory tract infections

Although the antibiotic prescription rate is high in absolute terms, supporting clinical information is required to better understand the quality of the prescribing. We found that two-thirds of all antibiotic use occurred in patients with a diagnosis of a respiratory tract infection (RTI), and that likely viral acute upper respiratory tract infections accounted for over 80% of the total antibiotic use for RTIs. This is despite there being very little evidence of benefit of antibiotic for such AURIs, and a considerable number of strong recommendations that they should be avoided.[22,23] The antibiotic prescribing rate for AURIs of 60% is similar to previous studies in rural village clinics in Shandong province,[8] Anhui province[24] and in Western China [16] as well as reports from rural India,[4,25] and Malaysia,[26] but far higher than rates observed in national-level studies in Sweden (8%), Belgium (19%) and Netherlands (38%).[27]

Antibiotic prescribing quality indicators were recently developed in Europe to assess both the decision to prescribe an antibiotic in specific clinical conditions, and the actual choice of antibiotic agent.[14] All of our results lay outside of the recommended ranges. In particular, we found that the recommended J01CE antibiotics were rarely used (1.5% for AURI, 2.9% for tonsillitis), whereas J01CA penicillins were used far more frequently. This may represent a prescriber preference for broader spectrum antibiotics.

Clinical treatment guidelines are not in widespread use in village clinics in China [28], however there are essential medicine lists and lists of antibiotics which require higher authorisation levels to be prescribed. All but one antibiotic prescribed for AURIs were either included on the Chinese national essential medicines list [29], or on the list of supplemental essential medicines available in Shandong province; cefixime did not feature on either of these two lists and accounted for 1.6% of all antibiotics prescribed for AURIs during the study period. The high rate of parenteral antibiotics use, third-generation cephalosporins and fluroquinolones in our study are concerning,

For peer review only

but are consistent with the results of province-wide antibiotic consumption data that were recently reported in Shandong province using procurement records,[30] as well as previous studies at healthcare institutions in rural Shandong province.[8] In a survey we previously conducted at the village clinics included in this study, 74% of doctors reported thinking that parenteral antibiotics are more effective than oral antibiotics.[31]

Interestingly, we found that antibiotics were used less frequently than recommended for adult patients with pneumonia (73.3% compared with a target of 90-100%),[14] potentially representing underuse of antibiotics. Analgesic and anti-inflammatory medicines are potentially under-used, since they have the potential to both provide symptomatic relief and satisfy patients' expectations to gain some form of medicine from visiting the doctor; fewer than half of all patients with a diagnosis of AURI received such a medication.

Variations in antibiotic prescribing between prescribers

Understanding what variations exist in clinical care, and why, is important for developing targets as well as context-specific interventions capable of reaching these targets. It is also essential for working towards individuals having equitable access to healthcare, irrespective of where they reside.

Our study design has enabled us to investigate variability in antibiotic prescribing at the prescriber-level whilst keeping several factors relatively constant: the villages served by the clinics are limited to a small geographical region and are quite homogeneous in terms of socio-economic levels; the patient populations in the villages are likely to have similar consultation behaviours when ill, in particular for a common diagnosis such as AURI; the doctors all have the same medical qualifications and at least 14 years of clinical experience; the village clinics are all subject to the same reimbursement systems (including zero mark-up on antibiotics) and regulations concerning antibiotic use.[32,33] Given these similarities, it might be hypothesised that little variation would exist in the prescribing practices between the doctors. Our results, however, revealed substantial differences between prescribers. Patients with AURI who were seen by the high-antibiotic prescribers were twice as likely to be given a prescription containing an antibiotic, four times as likely to be given two or more antibiotics, and three times as likely to receive at least one parenteral antibiotic. There was also significant variation within the high-prescriber group, highlighting the importance of individual-level analyses: patients with AURI who visited doctors 513 and 412 were equally likely to be prescribed an antibiotic (mean APR of 87%), but those who visited doctor 513 were six times more likely to be prescribed two or more antibiotics, three times more likely to be prescribed an parenteral antibiotic, and a third less likely to be prescribed an analgesic or anti-inflammatory medicine; furthermore, the choice of antibiotic prescribed was likely to be an aminoglycoside, a third generation cephalosporin or a fluoroquinolone, compared with amoxicillin, a first-generation cephalosporin, or a macrolide. Furthermore, a single doctor was responsible for a quarter of all amikacin use during the study period. Such extreme variation is important for individual patients in terms of risks of developing antibiotic-related complications and the impact of antibiotic use on their normal flora.[34] This ethical component should not be understated, and there is a growing trend for antibiotic stewardship to be viewed in terms of patient safety and broader responsibility.[35,36]

Relatively few published studies have assessed variations between prescribers concerning

For peer review only

antibiotic use in primary care settings, with most studies analysing aggregated data and presenting averages. In 2001, Akkerman *et al* investigated antibiotic prescribing rate for upper respiratory tract infections among 84 primary care practitioners in the Netherlands, finding that this ranged from 15 to 27%.[37] More recently, Grover *et al* examined antibiotic prescribing for RTIs at a single outpatient practice in the US and found variations depending on the provider type from 55% (residents) to 84% (nurse practitioners).[38] Jung *et al* found an interquartile range of 27% to 60% in antibiotic prescribing rates for RTIs among 109 prescribers in a large healthcare network in the US;[39] a study conducted in a separate US healthcare network of urgent care clinics found an interquartile range in antibiotic prescribing rates among physicians of 7% to 28% for respiratory encounters where antibiotics are not indicated.[40] A tempting justification against conducting such individual-level analyses is that it is difficult to account for the variation in patient case mix. This does indeed limit the ability to make comparisons, particularly between studies conducted in settings in which patients have very different consultation rates and co-morbidities, or in studies that look at overall antibiotic prescription rates rather than at a single diagnosis. However, there is growing evidence from these studies that differences in patient case mix may not explain a large amount of variability in prescribing patterns for antibiotics.[9,39] Furthermore, ignoring variations between individual prescribers causes us to subtly obscure the role (or even responsibility) of the individual decision-maker. Interestingly, the doctors in our study used between 11 and 21 different antibiotic agents for patients with AURI, demonstrating the heterogeneity even within an individual prescriber.

Future studies should explore why such variations exist, as well as what the consequences are for efforts to improve antibiotic use. For example, qualitative studies with individual prescribers could help understand why some doctors already have very low rates of parenteral and multi-antibiotic use, and why prescribers have used such a wide range of antibiotic agents for patients with a single diagnosis. It is possible that structural factors such medicine shortages might contribute to changes in antibiotics prescribed. We did find that high antibiotic AURI prescribers were more likely than low AURI prescribers to prescribe antibiotics for the group of URTI diagnoses that are most likely to have bacterial causes (but where the most common causes are still viral). One possible explanation is that these prescribers could be more risk averse, and so are more likely than other doctors to prescribe antibiotics in cases where the cause could be bacterial;[41] an alternative is that these prescribers are more willing than others to follow patient demand, particularly if this is generally high. Future studies could also explore the influence of peers within clinics; our results suggest there is some clustering of behaviours within certain village clinics.

Methodological considerations

Our study has several strengths, including the long length of data collection and the high number of prescriptions included by individual prescribers. Furthermore, almost all patients that visit doctors at a village clinic receive a prescription. A common limitation of studies using prescription data is the inability to verify diagnoses. To restrict the impact of this limitation we restricted the individual-level analyses to AURI diagnoses, but we cannot guarantee that misclassifications did not occur, particularly given there is currently no incentive for doctors to correctly classify diagnoses. AURIs accounted on average for 86% of respiratory tract infection diagnoses by individual doctors, but this ranged from 58% to 100% which strongly suggests that there were variations in how doctors classified different infections, and this warrants further investigation, particularly if diagnosis-based antibiotic prescribing targets are ever introduced. Indeed, a recent

For peer review only

study concluded that deficits in diagnostic knowledge are a major driver of unnecessary antibiotic prescriptions in clinics in rural China.[42] Additional limitations include an inability to account for variations in the workload of individual doctors, and to control for patient co-morbidities. Finally, only one diagnosis can be included per prescription on the electronic prescribing system. It is possible that some antibiotics prescribed for likely viral acute upper respiratory tract infections are actually treatment for other infections that patients presented with at the same time; however, doctors would usually choose to write the more severe diagnosis, so we believe this is likely only relevant to a very small number of cases and will not have significantly affected our main findings.

Conclusion

Together our results provide evidence that even in a small homogenous setting in rural China, variations in individual prescriber practices are significant, and they need to be accounted for in the development of targets and interventions to improve antibiotic use. We suspect this finding is likely to be generalizable to other areas in rural Shandong province as a minimum, but also to other areas in eastern rural China, where the healthcare system is very similar in terms of structure, regulations and staffing. We show that there is a need for all prescribers to reduce their antibiotic prescribing rate for AURIs, but the extent of this reduction varies widely between prescribers. For some prescribers, reducing the proportion of prescriptions that contain fluoroquinolones and third-generation cephalosporins may be more important goals, both for their patients and for public health. Finally, it is possible that interventions that incorporate individual-level feedback will have a more directed and effective impact on improving antibiotic use. A promising recent example in Anhui province, China, was able to provide “just-in-time” individualised information and feedback to doctors in village clinics and improved antibiotic prescribing practices.[43]

Acknowledgements

We acknowledge the support of the local Centre for Disease Control and Prevention in coordinating this study, as well as all students who contributed to the data collection. We would also like to thank Yuanyuan Wang for assisting with the coding of diagnoses according to ICD-10 standards. Finally, we are grateful for the efforts of the IMPACT-consortium: www.folkhalsomyndigheten.se/impact/.

Competing interests

None to declare.

Funding

This work was supported by the Swedish Research Council (grant number D0879801) and National Natural Science Foundation of China (grant number 81361138021).

Author contributions

All authors were involved in the conception, design and implementation of the project. YD and JY participated in the data collection. OJD and YD analysed and interpreted the data, and drafted the manuscript, with JY, QS and CSL providing critical input at all stages. All authors approved the final version of the manuscript.

Data sharing statement

For peer review only

The data sets generated and analysed during the current study are available on reasonable request from the corresponding author.

For peer review only

References

1 van de Sande-Bruinsma N, Grundmann H, Verloo D, *et al.* Antimicrobial drug use and resistance in Europe. *Emerg Infect Dis* 2008;14:1722–30.

2 Davey P, Marwick CA, Scott CL, *et al.* Interventions to improve antibiotic prescribing practices for hospital inpatients. *Cochrane Database Syst Rev* 2017;2:CD003543.

3 Tamma PD, Cosgrove SE. Antimicrobial stewardship. *Infect Dis Clin North Am* 2011;25:245–60.

4 Dehn Lunn A. Reducing inappropriate antibiotic prescribing in upper respiratory tract infection in a primary care setting in Kolkata, India. *BMJ Open Qual* 2018;7:e000217.

5 Wang J, Wang P, Wang X, *et al.* Use and prescription of antibiotics in primary health care settings in china. *JAMA Intern Med* 2014;174:1914–20.

6 Li J, Song X, Yang T, *et al.* A Systematic Review of Antibiotic Prescription Associated With Upper Respiratory Tract Infections in China. *Medicine (Baltimore)* 2016;95:e3587.

7 Jiang Q, Yu BN, Ying G, *et al.* Outpatient prescription practices in rural township health centers in Sichuan Province, China. *BMC Health Serv Res* 2012;12:324.

8 Sun Q, Dyar OJ, Zhao L, *et al.* Overuse of antibiotics for the common cold – attitudes and behaviors among doctors in rural areas of Shandong Province, China. *BMC Pharmacol Toxicol* 2015;16:6.

9 Pouwels KB, Dolk FCK, Smith DRM, *et al.* Explaining variation in antibiotic prescribing between general practices in the UK. *J Antimicrob Chemother* 2018;73:ii27–35.

10 Stålsby Lundborg C, Tamhankar AJ. Understanding and changing human behaviour--antibiotic mainstreaming as an approach to facilitate modification of provider and consumer behaviour. *Ups J Med Sci* 2014;119:125–33.

11 Asia Pacific Observatory on Health Systems and Policies. People's Republic of China Health System Review. Manila: WHO Regional Office for the Western Pacific 2015.

12 Sun Q, Wang Y, Hulth A, *et al.* Study protocol for One Health data collections, analyses and intervention of the Sino-Swedish integrated multisectoral partnership for antibiotic resistance containment (IMPACT). *BMJ Open* 2018;8:e017832.

13 WHO Collaborating Centre for Drug Statistics Methodology. ATC classification index with DDDs, 2018. https://www.whocc.no/atc_ddd_index/ (accessed 1 August 2018).

14 Adriaenssens N, Coenen S, Tonkin-Crine S, *et al.* European Surveillance of Antimicrobial Consumption (ESAC): disease-specific quality indicators for outpatient antibiotic prescribing. *BMJ Qual Saf* 2011;20:764–72.

15 Benjamini Y, Hochberg Y. Controlling the False Discovery Rate: A Practical and Powerful Approach to Multiple Testing. *J R Stat Soc Ser B* 1995;57:289–300.

16 Dong L, Yan H, Wang D. Antibiotic prescribing patterns in village health clinics across 10 provinces of Western China. *J Antimicrob Chemother* 2008;62:410–5.

17 Dolk FCK, Pouwels KB, Smith DRM, *et al.* Antibiotics in primary care in England: which antibiotics are prescribed and for which conditions? *J Antimicrob Chemother* 2018;73:ii2–10.

18 Xiao Y, Li L. The actions of China in containing antimicrobial resistance. In: AMR Control 2015. 2015:46–53. http://www.globalhealthdynamics.co.uk/wp-content/uploads/2015/05/07_Xiao-Li.pdf (accessed 1 Mar 2020).

19 National Health Commission of the People's Republic of China. Further Strengthening the Management of Clinical Application of Antibacterial Medicines. 2015.

http://www.nhc.gov.cn/xxgk/pages/viewdocument.jsp?dispatchDate=&staticUrl=/yzygj/s3593/201508/f0fdf1f52df14b87aa97be53819f1036.shtml (accessed 1 Mar 2020)

20 Yin X, Song F, Gong Y, *et al.* A systematic review of antibiotic utilization in China. *J Antimicrob Chemother* 2013;68:2445–52.

21 Liu C, Liu C, Wang D, *et al.* Determinants of antibiotic prescribing behaviors of primary care physicians in Hubei of China: a structural equation model based on the theory of planned behavior. *Antimicrob Resist Infect Control* 2019;8:23.

22 Harris AM, Hicks LA, Qaseem A, *et al.* Appropriate Antibiotic Use for Acute Respiratory Tract Infection in Adults: Advice for High-Value Care From the American College of Physicians and the Centers for Disease Control and Prevention. *Ann Intern Med* 2016;164:425.

23 Kenealy T, Arroll B. Antibiotics for the common cold and acute purulent rhinitis. *Cochrane Database Syst Rev* 2013;6:CD000247.

24 Chai J, Coope C, Cheng J, *et al.* Cross-sectional study of the use of antimicrobials following common infections by rural residents in Anhui, China. *BMJ Open* 2019;9:e024856.

25 Khare S, Purohit M, Sharma M, *et al.* Antibiotic Prescribing by Informal Healthcare Providers for Common Illnesses: A Repeated Cross-Sectional Study in Rural India. *Antibiotics* 2019;8:139.

26 Ab Rahman N, Teng CL, Sivasampu S. Antibiotic prescribing in public and private practice: a cross-sectional study in primary care clinics in Malaysia. *BMC Infect Dis* 2016;16:208.

27 Tyrstrup M, van der Velden A, Engstrom S, *et al.* Antibiotic prescribing in relation to diagnoses and consultation rates in Belgium, the Netherlands and Sweden: use of European quality indicators. *Scand J Prim Health Care* 2017;35:10–8.

28 Reynolds L, McKee M. Factors influencing antibiotic prescribing in China: An exploratory analysis. *Health Policy (New York)* 2009;90:32–6.

29 National Health Commission of China. National Essential Medicines List (2018 edition). 2018. http://www.nhc.gov.cn/ewebeditor/uploadfile/2018/10/20181025183346942.pdf (accessed 1 Mar 2020).

30 Yin J, Li Q, Sun Q. Antibiotic consumption in Shandong Province, China: an analysis of provincial pharmaceutical centralized bidding procurement data at public healthcare institutions, 2012–16. *J Antimicrob Chemother* 2018;73:814–20.

31 Yin J, Dyar OJ, Yang P, *et al.* Pattern of antibiotic prescribing and factors associated with it in eight village clinics in rural Shandong Province, China: a descriptive study. *Trans R Soc Trop Med Hyg* 2019;113:714–21.

32 Yi H, Miller G, Zhang L, *et al.* Intended and unintended consequences of China's zero markup drug policy. *Health Aff (Millwood)* 2015;34:1391–8.

33 Xiao Y, Li L. Legislation of clinical antibiotic use in China. *Lancet Infect Dis* 2013;13:189–91.

34 Costelloe C, Metcalfe C, Lovering A, *et al.* Effect of antibiotic prescribing in primary care on antimicrobial resistance in individual patients: systematic review and meta-analysis. *BMJ* 2010;340:c2096.

35 Tamma PD, Holmes A, Ashley ED. Antimicrobial stewardship. *Curr Opin Infect Dis* 2014;27:348–55.

36 Dyar OJ, Obua C, Chandy S, *et al.* Using antibiotics responsibly: are we there yet? *Future Microbiol* 2016;11:1057–71.

37 Akkerman AE, Kuyvenhoven MM, van der Wouden JC, *et al.* Prescribing antibiotics for respiratory tract infections by GPs: management and prescriber characteristics. *Br J Gen Pract* 2005;55:114–8.

For peer review only

38 Grover ML, Mookadam M, Rutkowski RH, *et al.* Acute respiratory tract infection: a practice examines its antibiotic prescribing habits. *J Fam Pract* 2012;61:330–5.

39 Jung S, Sexton ME, Owens S, *et al.* Variability of Antibiotic Prescribing in a Large Healthcare Network Despite Adjusting for Patient-Mix: Reconsidering Targets for Improved Prescribing. *Open forum Infect Dis* 2019;6:ofz018.

40 Stenehjem E, Wallin A, Fleming-Dutra KE, *et al.* Antibiotic Prescribing Variability in a Large Urgent Care Network: A New Target for Outpatient Stewardship. *Clin Infect Dis* Published Online First: 23 October 2019. doi:10.1093/cid/ciz910

41 Deschepper R, Grigoryan L, Lundborg CS, *et al.* Are cultural dimensions relevant for explaining cross-national differences in antibiotic use in Europe? *BMC Health Serv Res* 2008;8:123.

42 Xue H, Shi Y, Huang L, *et al.* Diagnostic ability and inappropriate antibiotic prescriptions: a quasi-experimental study of primary care providers in rural China. *J Antimicrob Chemother* 2019;74:256–63.

43 Shen X, Lu M, Feng R, *et al.* Web-Based Just-in-Time Information and Feedback on Antibiotic Use for Village Doctors in Rural Anhui, China: Randomized Controlled Trial. *J Med Internet Res* 2018;20:e53.

For peer review only

FIGURE LEGENDS

Figure 1. Medicine types prescribed for AURIs

Figure footnote: Analgesics or anti-inflammatories = any of the following: aspirin/acetylsalicylic acid, ibuprofen, paracetamol or diclofenac.

Figure 2. Antibiotic prescribing rates for likely viral AURIs for individual prescribers

Figure footnote: AURI = likely viral acute upper respiratory tract infections

Figure 3. Different medicine types used for likely viral AURIs by individual prescribers

For peer review only

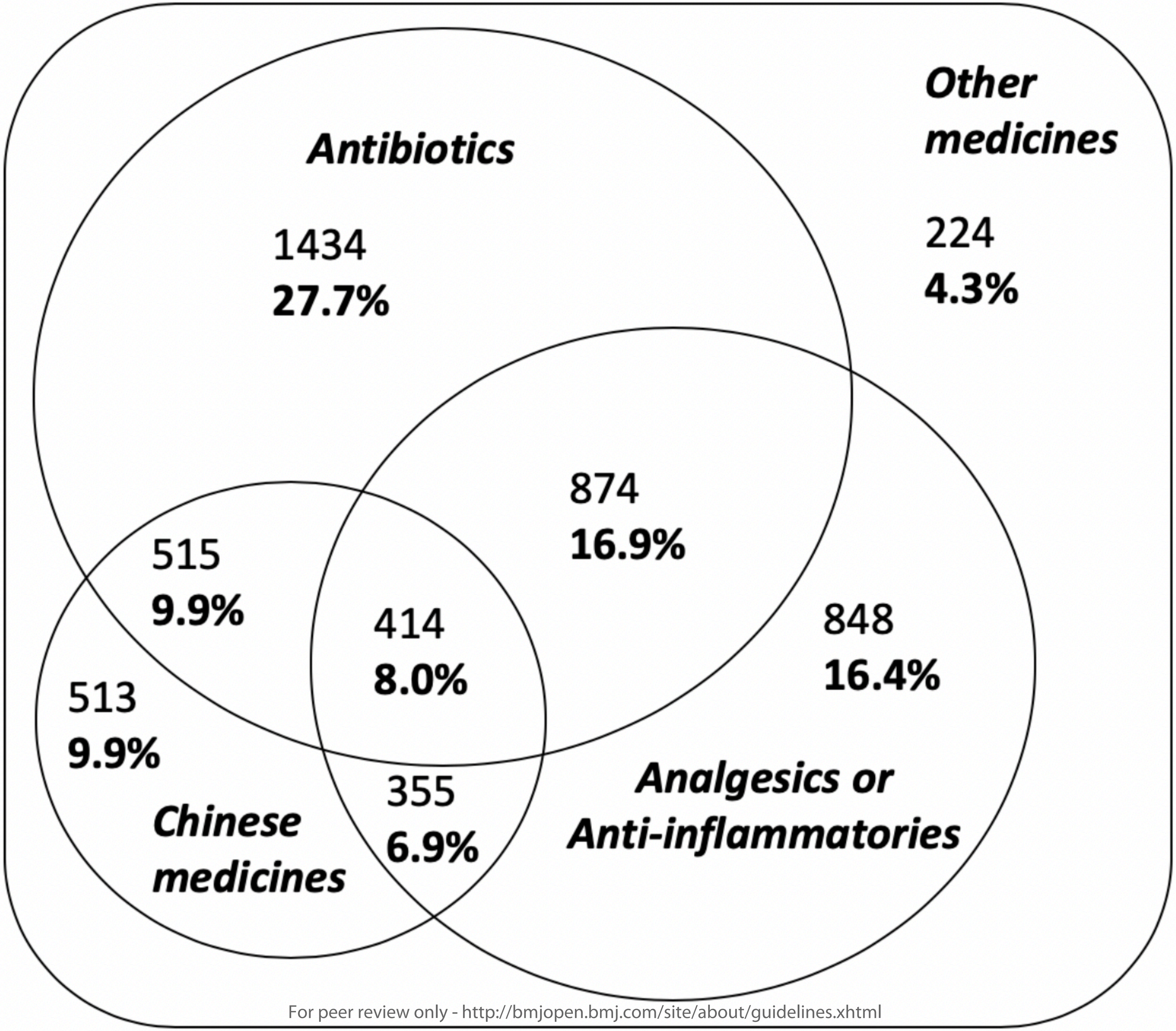
Antibiotics
Other medicines
1434
27.7%
224
4.3%
874
16.9%
515
9.9%
414
8.0%
848
16.4%
513
9.9%
355
6.9%
Chinese medicines
Analgesics or Anti-inflammatories

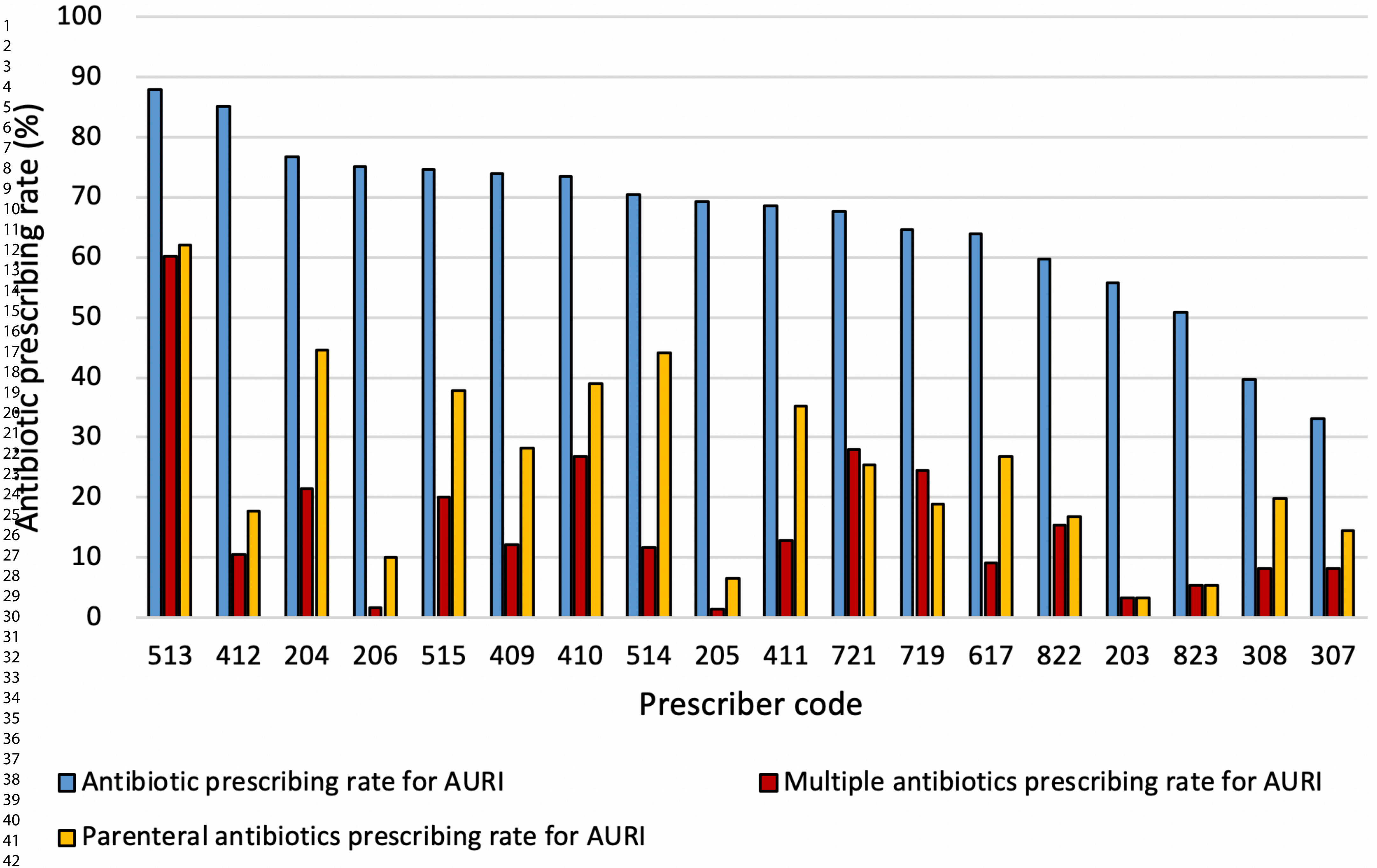

Antibiotic prescribing rate (%)
100
90
80
70
60
50
40
30
20
10
0
513
412
204
206
515
409
410
514
205
411
721
719
617
822
203
823
308
307
Prescriber code
Antibiotic prescribing rate for AURI
Multiple antibiotics prescribing rate for AURI
Parenteral antibiotics prescribing rate for AURI

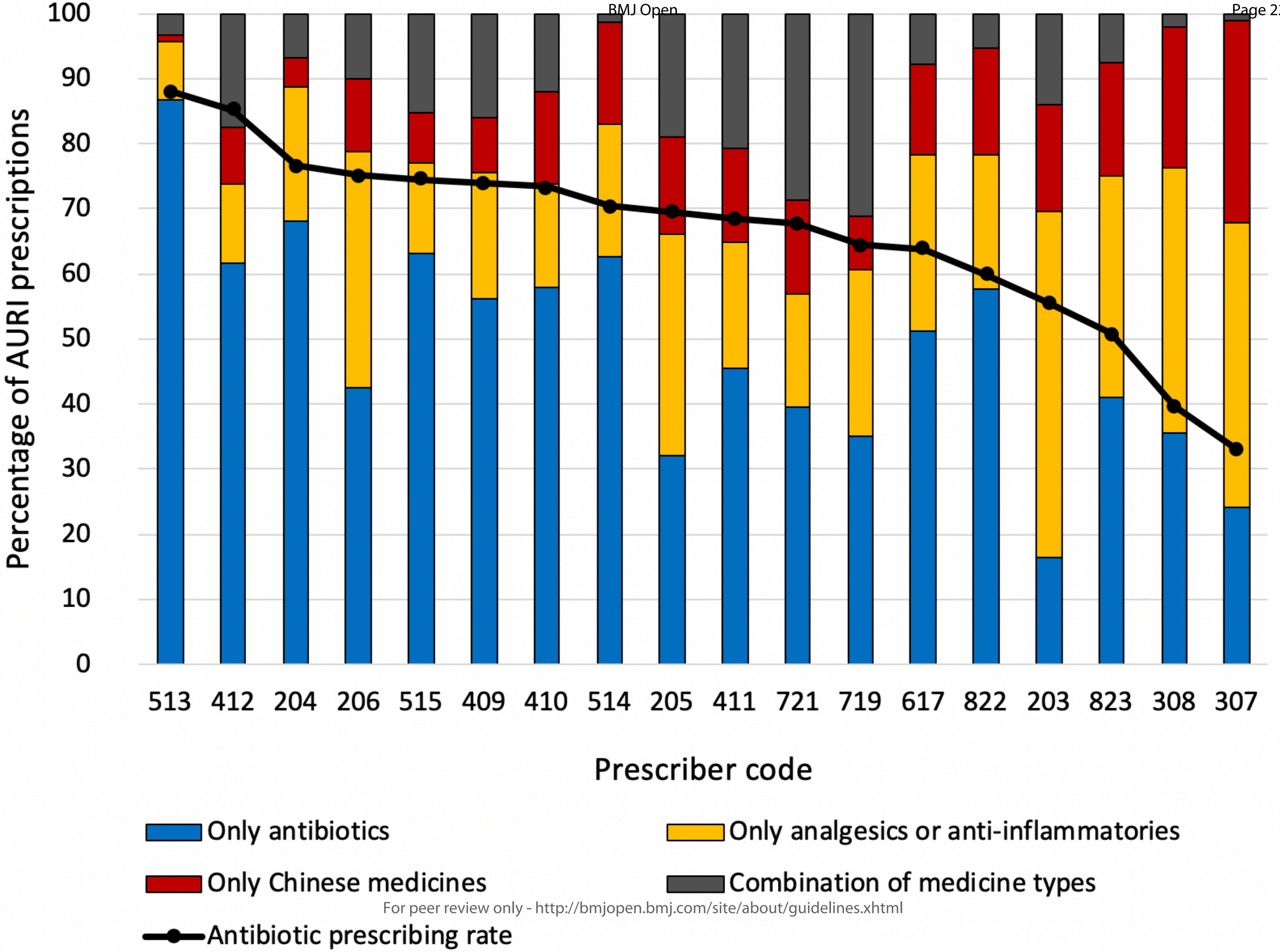
100
90
80
70
60
50
40
30
20
10
0
Percentage of AURI prescriptions
513
412
204
206
515
409
410
514
205
411
721
719
617
822
203
823
308
307
Prescriber code
Only antibiotics
Only analgesics or anti-inflammatories
Only Chinese medicines
Combination of medicine types
Antibiotic prescribing rate

Supplementary tables for:

Variations in antibiotic prescribing among village doctors in a rural region of Shandong province, China: a cross-sectional analysis of prescriptions

Oliver J. Dyar, Yang Ding, Jia Yin, Sun Qiang+, Cecilia Stålsby Lundborg

+Corresponding author: School of Health Care Management, Shandong University, Jinan, Shandong, China. Tel: +86-531-88382376; Fax: +86-531-88382693; E-mail: qiangs@sdu.edu.cn

For peer review only

Appendix table 1. Class and agent of antibiotic prescribing for AURI (N = 3818)

Antibiotic class	Frequency	%	Antibiotic agent	Frequency	%
J01AA Tetracyclines	3	0.1	J01AA02 Doxycycline	3	0.1
J01CA Penicillins with Extended Spectrum	***1025***	***26.8***	J01CA01 Ampicillin	8	0.2
			J01CA04 Amoxicillin	***1017***	***26.6***
J01CE Beta-Lactamase Sensitive Penicillins	49	1.3	J01CE01 Benzylpenicillin	35	0.9
			J01CE02 Phenoxymethylpenicillin	14	0.4
J01CR Combinations of Penicillins, Incl. Beta-Lactamase Inhibitors	79	2.1	J01CR02 Amoxicillin and enzyme inhibitor	79	2.1
J01DB First-Generation Cephalosporins	***603***	***15.8***	***J01DB01 Cefalexin***	***323***	***8.5***
			J01DB09 Cefradine	280	7.3
J01DC Second-Generation Cephalosporins	***272***	***7.1***	J01DC02 Cefuroxime	272	7.1
J01DD Third-Generation Cephalosporins	255	6.7	J01DD01 Cefotaxime	193	5.1
			J01DD08 Cefixime	62	1.6
J01EE Combinations of Sulfonamides and Trimethoprim, Incl. Derivatives	1	0.0	J01EE01 Sulfamethoxazole	1	0.0
J01FA Macrolides	***516***	***13.5***	***J01FA06 Roxithromycin***	***317***	***8.3***
			J01FA09 Clarithromycin	7	0.2
			J01FA10 Azithromycin	192	5.0
J01FF Lincosamides	343	9.0	J01FF01 Clindamycin	45	1.2
			J01FF02 Lincomycin	***298***	***7.8***
J01GB Other Aminoglycosides	166	4.3	J01GB03 Gentamicin	37	1.0
			J01GB06 Amikacin	129	3.4
J01MA Fluoroquinolones	***423***	***11.1***	J01MA02 Ciprofloxacin	64	1.7
			J01MA06 Norfloxacin	25	0.7
			J01MA12 Levofloxacin	***334***	***8.7***
J01MB Other Quinolones	20	0.5	J01MB04 Pipemidic Acid	20	0.5
J01XD Imidazole Derivatives	58	1.5	J01XD01 Metronidazole	8	0.2
			J01XD02 Tinidazole	50	1.3
J01XE Nitrofuran Derivatives	3	0.1	J01XE01 Nitrofurantoin	3	0.1
J01XX Other Antibacterials	2	0.1	J01XX01 Fosfomycin	2	0.1

For peer review only

Appendix Table 2. Additional antibiotic prescribing rate indicators for individual prescribers

Prescriber	Clinic	Sex	Age	% of respiratory tract infection diagnoses coded as AURI			APR of AURI in 2015			APR of AURI in 2016			APR of potentially bacterial URTIs			APR of gastroenteritis, diarrhoea and colitis		
				n	N	%	n	N	%	n	N	%	n	N	%	n	N	%
101	BFZG	2	46	377	647	58.3	151	181	83.4	70	142	49.3	56	61	91.8	58	62	93.5
102	BFZG	1	52	56	76	73.7	NA	NA	NA	NA	NA	NA	9	9	100.0	NA	NA	NA
203	CS	1	59	167	251	66.5	12	26	46.2	40	71	56.3	63	83	75.9	2	2	100.0
204	CS	1	46	73	126	57.9	17	22	77.3	21	28	75.0	50	52	96.2	10	10	100.0
205	CS	2	50	111	141	78.7	37	63	58.7	19	24	79.2	20	27	74.1	3	3	100.0
206	CS	1	63	173	216	80.1	57	87	65.5	73	86	84.9	32	39	82.1	10	10	100.0
307	DS	2	40	520	605	86.0	88	199	44.2	38	160	23.8	39	51	76.5	64	111	57.7
308	DS	1	67	217	251	86.5	57	132	43.2	29	84	34.5	20	28	71.4	33	70	47.1
409	FJZZ	1	63	134	136	98.5	54	77	70.1	45	57	78.9	NA	NA	NA	21	46	45.7
410	FJZZ	1	40	199	200	99.5	82	123	66.7	64	76	84.2	NA	NA	NA	25	48	52.1
411	FJZZ	1	43	124	146	84.9	NA	NA	NA	26	38	68.4	7	9	77.8	12	15	80.0
412	FJZZ	1	60	271	285	95.1	98	108	90.7	92	118	78.0	2	2	100.0	38	69	55.1
513	QD	1	46	117	124	94.4	76	89	85.4	27	28	96.4	NA	NA	NA	4	4	100.0
514	QD	1	52	578	597	96.8	113	123	91.9	168	222	75.7	2	2	100.0	31	35	88.6
515	QD	1	65	181	192	94.3	80	102	78.4	55	79	69.6	NA	NA	NA	16	20	80.0
616	XJG	1	63	19	33	57.6	4	14	28.6	1	5	20.0	6	9	66.7	2	3	66.7
617	XJG	1	39	415	546	76.0	76	117	65.0	98	163	60.1	22	31	71.0	69	100	69.0
618	XJG	1	37	12	13	92.3	NA	NA	NA	8	12	66.7	0	1	0.0	12	17	70.6
719	YJ	1	42	386	437	88.3	104	153	68.0	78	129	60.5	1	1	100.0	33	38	86.8
720	YJ	1	63	3	3	100.0	0	1	0.0	1	2	50.0	NA	NA	NA	NA	NA	NA
721	YJ	1	36	408	416	98.1	114	152	75.0	108	163	66.3	1	1	100.0	30	35	85.7
822	YJZZ	1	68	378	407	92.9	127	184	69.0	99	194	51.0	9	20	45.0	46	50	92.0
823	YJZZ	2	55	258	335	77.0	69	95	72.6	30	67	44.8	23	35	65.7	38	38	100.0

Footnote: APR = antibiotic prescribing rate; AURI = likely viral acute upper respiratory tract infections; potentially bacterial upper respiratory tract infections (URTIs) = prescription containing and diagnosis of pharyngitis, tonsillitis or laryngopharyngitis. Rows in grey are not included in the individual prescriber-level analyses in the main text due to contributing <50 AURI prescriptions (prescribers 616, 618, 720) and due to an error in the coding of prescriber (prescribers 101, 102). 1 = male; 2 = female.

For peer review only

Appendix table 3. Most common antibiotic agents prescribed for AURI at individual prescriber level

Prescriber	Clinic	Sex	Age	1st most common antibiotic prescribed for AURI, name (no.)			2nd most common antibiotic prescribed for AURI, name (no.)			3rd most common antibiotic prescribed for AURI, name (no.)			Sum top 3	Total no. abx	% abx that are top three	No. abx agents total
101	BFZG	2	46	J01CA04	Amoxicillin	126	J01DB01	Cefalexin	35	J01FA06	Roxithromycin	31	192	247	77.7	13
102	BFZG	1	52	J01CA04	Amoxicillin	9							9	9	100.0	1
203	CS	1	59	J01MA12	Levofloxacin	25	J01DB09	Cefradine	21	J01CA04	Amoxicillin	16	62	93	66.7	13
204	CS	1	46	J01MA12	Levofloxacin	16	J01CA04	Amoxicillin	13	J01DD01	Cefotaxime	12	41	56	73.2	11
205	CS	2	50	J01CA04	Amoxicillin	21	J01DB09	Cefradine	12	J01MA12	Levofloxacin	12	45	77	58.4	12
206	CS	1	63	J01CA04	Amoxicillin	46	J01DB09	Cefradine	23	J01DB01	Cefalexin	19	88	130	67.7	11
307	DS	2	40	J01CA04	Amoxicillin	35	J01MA02	Ciprofloxacin	23	J01FA06	Roxithromycin	20	78	172	45.3	20
308	DS	1	67	J01CA04	Amoxicillin	24	J01DB01	Cefalexin	15	J01DB09<br>J01DC02<br>J01MA02	Cefradine<br>Cefuroxime<br>Ciprofloxacin	9<br>9<br>9	48	86	55.8	17
409	FJZZ	1	63	J01CA04	Amoxicillin	20	J01DB09	Cefradine	17	J01FA06	Roxithromycin	16	53	99	53.5	17
410	FJZZ	1	40	J01CA04	Amoxicillin	25	J01FA06	Roxithromycin	23	J01FF02	Lincomycin	23	71	146	48.6	18
411	FJZZ	1	43	J01FF02	Lincomycin	22	J01CA04	Amoxicillin	18	J01MA12	Levofloxacin	12	52	85	61.2	11
412	FJZZ	1	60	J01CA04	Amoxicillin	60	J01DB09	Cefradine	46	J01FA06	Roxithromycin	39	145	231	62.8	16
513	QD	1	46	J01GB06	Amikacin	33	J01DD01	Cefotaxime	28	J01MA12	Levofloxacin	28	89	103	86.4	16
514	QD	1	52	J01CA04	Amoxicillin	140	J01FF02	Lincomycin	137	J01DB09	Cefradine	28	305	407	74.9	21
515	QD	1	65	J01CA04	Amoxicillin	53	J01FF02	Lincomycin	27	J01GB06	Amikacin	15	95	135	70.4	18
616	XJG	1	63	J01CA04	Amoxicillin	1	J01CR02	Amoxicillin and enzyme inhibitor	1	J01DB01	Cefalexin	1	3	5	60.0	6
617	XJG	1	39	J01FA10	Azithromycin	49	J01CA04	Amoxicillin	46	J01FA06	Roxithromycin	42	137	265	51.7	19
618	XJG	1	37	J01CA04	Amoxicillin	2	J01DC02	Cefuroxime	2	J01DB09	Cefradine	1	5	8	62.5	7
719	YJ	1	42	J01CA04	Amoxicillin	86	J01DC02	Cefuroxime	43	J01DB01	Cefalexin	39	168	249	67.5	20
720	YJ	1	63	J01GB06	Amikacin	1	J01AA02						1	1	100.0	1
721	YJ	1	36	J01CA04	Amoxicillin	68	J01MA12	Levofloxacin	59	J01FA06	Roxithromycin	43	170	276	61.6	20
822	YJZZ	1	68	J01CA04	Amoxicillin	112	J01FA06	Roxithromycin	26	J01DC02	Cefuroxime	25	163	226	72.1	17

823	YJZZ	2	55	J01CA04	Amoxicillin	75	J01DB09	Cefradine	18	J01DB01	Cefalexin	14	107	131	81.7	13

Footnote: AURI = likely viral acute upper respiratory tract infections; abx = antibiotics; 1 = male; 2 = female. Rows in grey are not included in the individual prescriber-level analyses in the main text due to contributing <50 AURI prescriptions (prescribers 616, 618, 720) and due to an error in the coding of prescriber (prescribers 101, 102).

For peer review only

Appendix table 4. Antibiotic use for AURI at level of individual prescriber

Prescriber	Clinic	Sex	Age	Patient age			Female patients		APR for ALL prescriptions			APR for AURI			MPR for AURI			PAPR for AURI			AURI with analgesic or anti-inflammatory		
				Mean	Min	Max	n	%	n	N	%	n	N	%	n	N	%	n	N	%	n	N	%
101	BFZG	2	46	48	2	90	152	40.3	794	1674	47.4	247	377	65.5	24	247	9.7	10	247	4.0	260	377	69.0
102	BFZG	1	52	50	13	78	19	33.9	37	185	20.0	9	56	16.1	0	9	0.0	0	9	0.0	35	56	62.5
203	CS	1	59	53	5	81	53	31.7	226	598	37.8	93	167	55.7	3	93	3.2	3	93	3.2	127	167	76.0
204	CS	1	46	56	36	75	37	50.7	165	283	58.3	56	73	76.7	12	56	21.4	25	56	44.6	34	73	46.6
205	CS	2	50	52	9	80	50	45.0	142	357	39.8	77	111	69.4	1	77	1.3	5	77	6.5	66	111	59.5
206	CS	1	63	49	5	86	62	35.8	246	560	43.9	130	173	75.1	2	130	1.5	13	130	10.0	109	173	63.0
307	DS	2	40	50	1	90	206	39.6	372	1255	29.6	172	520	33.1	14	172	8.1	25	172	14.5	229	520	44.0
308	DS	1	67	47	4	89	95	43.8	171	576	29.7	86	217	39.6	7	86	8.1	17	86	19.8	78	217	35.9
409	FJZZ	1	63	54	5	84	64	47.8	138	328	42.1	99	134	73.9	12	99	12.1	28	99	28.3	55	134	41.0
410	FJZZ	1	40	53	3	86	95	47.7	191	405	47.2	146	199	73.4	39	146	26.7	57	146	39.0	80	199	40.2
411	FJZZ	1	43	56	10	83	57	46.0	144	365	39.5	85	124	68.5	11	85	12.9	30	85	35.3	61	124	49.2
412	FJZZ	1	60	54	5	90	137	50.6	329	654	50.3	231	271	85.2	24	231	10.4	41	231	17.7	134	271	49.4
513	QD	1	46	47	10	85	57	48.7	138	197	70.1	103	117	88.0	62	103	60.2	64	103	62.1	20	117	17.1
514	QD	1	52	49	2	92	286	49.5	528	1111	47.5	407	578	70.4	47	407	11.5	179	407	44.0	205	578	35.5
515	QD	1	65	49	3	83	86	47.5	196	417	47.0	135	181	74.6	27	135	20.0	51	135	37.8	83	181	45.9
616	XJG	1	63	56	5	82	4	21.1	34	101	33.7	5	19	26.3	1	5	20.0	2	5	40.0	7	19	36.8
617	XJG	1	39	44	1	87	156	37.6	536	1634	32.8	265	415	63.9	24	265	9.1	71	265	26.8	173	415	41.7
618	XJG	1	37	44	10	63	4	33.3	27	91	29.7	8	12	66.7	1	8	12.5	1	8	12.5	6	12	50.0
719	YJ	1	42	54	2	90	178	46.1	416	923	45.1	249	386	64.5	61	249	24.5	47	249	18.9	225	386	58.3
720	YJ	1	63	60	34	73	3	100.0	1	5	20.0	1	3	33.3	0	1	0.0	1	1	100.0	2	3	66.7
721	YJ	1	36	57	4	91	150	36.8	415	928	44.7	276	408	67.6	77	276	27.9	70	276	25.4	202	408	49.5
822	YJZZ	1	68	50	2	87	171	45.2	338	1038	32.6	226	378	59.8	35	226	15.5	38	226	16.8	159	378	42.1
823	YJZZ	2	55	48	2	86	119	46.1	249	786	31.7	131	258	50.8	7	131	5.3	7	131	5.3	141	258	54.7

Footnote: APR = antibiotic prescribing rate; MPR = multiple antibiotic prescribing rate; PAPR = parenteral antibiotic prescribing rate; AURI = likely viral acute upper respiratory tract infections; 1 = male; 2 = female. Rows in grey are not included in the individual prescriber-level analyses in the main text due to contributing <50 AURI prescriptions (prescribers 616, 618, 720) and due to an error in the coding of prescriber (prescribers 101, 102).

For peer review only - http://bmjopen.bmj.com/site/about/guidelines.xhtml

Appendix Table 5. Different types of medicines used by individual prescribers for AURI

Prescriber	Clinic	Sex	Age	AURI rx	Only abx		Only inf		Only TCM		Abx plus inf		Abx plus TCM		Inf plus TCM		Abx plus inf plus TCM		Only other drugs		Only abx/ only inf
				n	n	%	n	%	n	%	n	%	n	%	n	%	n	%	n	%	Ratio
101	BFZG	2	46	377	19	5.0	26	6.9	29	7.7	70	18.6	65	17.2	71	18.8	93	24.7	4	1.1	0.7
102	BFZG	1	52	56	0	0.0	30	53.6	17	30.4	5	8.9	4	7.1	0	0	0	0	0	0	0
203	CS	1	59	167	13	7.8	42	25.1	13	7.8	61	36.5	8	4.8	13	7.8	11	6.6	6	3.6	0.3
204	CS	1	46	73	30	41.1	9	12.3	2	2.7	19	26	4	5.5	3	4.1	3	4.1	3	4.1	3.3
205	CS	2	50	111	17	15.3	18	16.2	8	7.2	33	29.7	17	15.3	5	4.5	10	9	3	2.7	0.9
206	CS	1	63	173	34	19.7	29	16.8	9	5.2	69	39.9	19	11	3	1.7	8	4.6	2	1.2	1.2
307	DS	2	40	520	92	17.7	167	32.1	118	22.7	41	7.9	35	6.7	17	3.3	4	0.8	46	8.8	0.6
308	DS	1	67	217	54	24.9	62	28.6	33	15.2	10	4.6	19	8.8	3	1.4	3	1.4	33	15.2	0.9
409	FJZZ	1	63	134	46	34.3	16	11.9	7	5.2	22	16.4	18	13.4	4	3	13	9.7	8	6	2.9
410	FJZZ	1	40	199	73	36.7	20	10.1	18	9.0	36	18.1	22	11.1	9	4.5	15	7.5	6	3	3.7
411	FJZZ	1	43	124	35	28.2	15	12.1	11	8.9	19	15.3	15	12.1	11	8.9	16	12.9	2	1.6	2.3
412	FJZZ	1	60	271	92	33.9	18	6.6	13	4.8	82	30.3	31	11.4	8	3	26	9.6	1	0.4	5.1
513	QD	1	46	117	79	67.5	8	6.8	1	0.9	9	7.7	12	10.3	0	0	3	2.6	5	4.3	9.9
514	QD	1	52	578	264	45.7	86	14.9	65	11.2	100	17.3	37	6.4	13	2.2	6	1	7	1.2	3.1
515	QD	1	65	181	58	32.0	13	7.2	7	3.9	40	22.1	23	12.7	16	8.8	14	7.7	10	5.5	4.5
616	XJG	1	63	19	3	15.8	5	26.3	6	31.6	1	5.3	1	5.3	1	5.3	0	0	2	10.5	0.6
617	XJG	1	39	415	156	37.6	83	20.0	42	10.1	55	13.3	30	7.2	11	2.7	24	5.8	14	3.4	1.9
618	XJG	1	37	12	4	33.3	3	25.0	0	0.0	3	25	1	8.3	0	0	0	0	1	8.3	1.3
719	YJ	1	42	386	83	21.5	61	15.8	19	4.9	42	10.9	50	13	48	12.4	74	19.2	9	2.3	1.4
720	YJ	1	63	3	1	33.3	1	33.3	0	0.0	0	0	0	0	1	33.3	0	0	0	0	1
721	YJ	1	36	408	94	23.0	41	10.0	34	8.3	62	15.2	52	12.7	31	7.6	68	16.7	26	6.4	2.3
822	YJZZ	1	68	378	128	33.9	46	12.2	36	9.5	52	13.8	34	9	49	13	12	3.2	21	5.6	2.8
823	YJZZ	2	55	258	59	22.9	49	19.0	25	9.7	43	16.7	18	7	38	14.7	11	4.3	15	5.8	1.2

Footnote: rx = prescriptions; abx = antibiotic; inf = analgesic or anti-inflammatory medicines (any of the following: aspirin/acetylsalicylic acid, ibuprofen, paracetamol or diclofenac); TCM = traditional Chinese medicine; 1 = male; 2 = female. Rows in grey are not included in the individual prescriber-level analyses in the main text due to contributing <50 AURI prescriptions (prescribers 616, 618, 720) and due to an error in the coding of prescriber (prescribers 101, 102).

Supplementary material: STROBE Statement—checklist of items that should be included in reports of observational studies, for: Variations in antibiotic prescribing among village doctors in a rural region of Shandong province, China: a cross-sectional analysis of prescriptions

Oliver J. Dyar, Yang Ding, Jia Yin, Sun Qiang[+], Cecilia Stålsby Lundborg

[+]Corresponding author: School of Health Care Management, Shandong University, Jinan, Shandong, China. Tel: +86-531-88382376; Fax: +86-531-88382693; E-mail: qiangs@sdu.edu.cn

	Item No.	Recommendation	Page No.	Relevant text from manuscript
**Title and abstract**	1	(*a*) Indicate the study's design with a commonly used term in the title or the abstract	1	"Variations in antibiotic prescribing among village doctors in a rural region of Shandong province, China: a cross-sectional analysis of prescriptions"
		(*b*) Provide in the abstract an informative and balanced summary of what was done and what was found	2	As per abstract
**Introduction**				
Background/rationale	2	Explain the scientific background and rationale for the investigation being reported	3	e.g. "These studies have not been capable of investigating the extent of variability in practices that exist"
Objectives	3	State specific objectives, including any prespecified hypotheses	3	e.g. "Our aims were to assess if there was overall evidence of irrational antibiotic use, and whether there was significant variability between the practices of individual prescribers in this small, homogenous region."

For peer review only - http://bmjopen.bmj.com/site/about/guidelines.xhtml

**Methods**				
Study design	4	Present key elements of study design early in the paper	3	e.g. "We conducted a prospective observational analysis of outpatient prescriptions from January 2015 to July 2017 at eight village clinics clustered around a single town in Z County, Shandong province."
Setting	5	Describe the setting, locations, and relevant dates, including periods of recruitment, exposure, follow-up, and data collection	3-4	e.g. sections on study design and setting, data collection
Participants	6	(*a*) *Cohort study*—Give the eligibility criteria, and the sources and methods of selection of participants. Describe methods of follow-up<br>*Case-control study*—Give the eligibility criteria, and the sources and methods of case ascertainment and control selection. Give the rationale for the choice of cases and controls<br>*Cross-sectional study*—Give the eligibility criteria, and the sources and methods of selection of participants	3-4	e.g. description of how village clinics were selected as part of broader IMPACT research programme
		(*b*) *Cohort study*—For matched studies, give matching criteria and number of exposed and unexposed<br>*Case-control study*—For matched studies, give matching criteria and the number of controls per case	Not relevant	
Variables	7	Clearly define all outcomes, exposures, predictors, potential confounders, and effect modifiers. Give diagnostic criteria, if applicable	4-5	e.g. descriptions of coding of prescriptions; definitions of antibiotic prescribing rate, multiple antibiotics prescribing rate, parenteral antibiotic prescribing rate; European quality indicators comparison
Data sources/ measurement	8*	For each variable of interest, give sources of data and details of methods of assessment (measurement). Describe comparability of assessment methods if there is more than one group	4	e.g. descriptions of sampling of prescriptions under "data collection" and also data

For peer review only - http://bmjopen.bmj.com/site/about/guidelines.xhtml

				management
Bias	9	Describe any efforts to address potential sources of bias	4	e.g. "Prescriber-level analyses were limited to the 20/23 doctors with ≥50 AURI prescriptions during the study period, to ensure that the antibiotic prescribing rates calculated for each individual doctor were sufficiently representative of their practice."
Study size	10	Explain how the study size was arrived at	4	e.g. descriptions of how village clinics were selected as part of broader IMPACT research programme
Quantitative variables	11	Explain how quantitative variables were handled in the analyses. If applicable, describe which groupings were chosen and why	4	e.g. "A category of *likely viral acute upper respiratory tract infections* (AURI) was created by grouping diagnoses of J00 (acute nasopharyngitis [common cold]) and J06.9 (acute upper respiratory infection, unspecified)."
Statistical methods	12	(*a*) Describe all statistical methods, including those used to control for confounding	4-5	e.g. section on data management and analyses
		(*b*) Describe any methods used to examine subgroups and interactions	4-5	e.g. "Comparisons were also made against antibiotic prescribing quality indicators developed in Europe[14] for a subset of clinical diagnoses and patient ages."
		(*c*) Explain how missing data were addressed	4	e.g. "Prescriptions from one doctor who did not complete the

For peer review only - http://bmjopen.bmj.com/site/about/guidelines.xhtml

For peer review only

				questionnaire were removed (55 in total)."
		(*d*) *Cohort study*—If applicable, explain how loss to follow-up was addressed<br>*Case-control study*—If applicable, explain how matching of cases and controls was addressed<br>*Cross-sectional study*—If applicable, describe analytical methods taking account of sampling strategy	Not relevant	
		(*e*) Describe any sensitivity analyses	Not conducted	
**Results**				
Participants	13*	(a) Report numbers of individuals at each stage of study—eg numbers potentially eligible, examined for eligibility, confirmed eligible, included in the study, completing follow-up, and analysed	5	e.g. "In total 14471 prescriptions from 23 prescribers from January 2015 to July 2017 were included in the analyses. This included an average of 58 prescriptions per month per village clinic (mean range from 47 to 62).". Additional text in methods and in discussion is relevant.
		(b) Give reasons for non-participation at each stage	4	e.g. "Of the three doctors with fewer than 50 AURI prescriptions, two retired from clinical practice near the beginning of the study period (616 and 720), and one newly started working at the specific village clinic at the end of the study period (618)."
		(c) Consider use of a flow diagram	Not sufficiently valuable	
Descriptive data	14*	(a) Give characteristics of study participants (eg demographic, clinical, social) and information on exposures and potential confounders	7-8	E.g. section on "prescriber demographics" and Table 3

For peer review only - http://bmjopen.bmj.com/site/about/guidelines.xhtml

		(b) Indicate number of participants with missing data for each variable of interest	Not relevant	
		(c) *Cohort study*—Summarise follow-up time (eg, average and total amount)	Not relevant	
Outcome data	15*	*Cohort study*—Report numbers of outcome events or summary measures over time		
		Case-control study—Report numbers in each exposure category, or summary measures of exposure		
		Cross-sectional study—Report numbers of outcome events or summary measures	5-6	e.g. "Overall, 40.3% of the prescriptions (5833/14471) contained at least one antibiotic." Also, table 1.
Main results	16	(*a*) Give unadjusted estimates and, if applicable, confounder-adjusted estimates and their precision (eg, 95% confidence interval). Make clear which confounders were adjusted for and why they were included	5-9	e.g. "Overall, respiratory tract infections accounted for 68.4% (3991/5833) and gastrointestinal conditions for 14.0% (815/5833) of all prescriptions containing at least one antibiotic.", and several other results. Due to the small number of prescribers, ranges in prescribing rates are provided, for example in section on "Variations at the prescriber-level" and Figure 2.
		(*b*) Report category boundaries when continuous variables were categorized	Not relevant	
		(*c*) If relevant, consider translating estimates of relative risk into absolute risk for a meaningful time period	Not relevant	
Other analyses	17	Report other analyses done—eg analyses of subgroups and interactions, and sensitivity analyses	7, 9	e.g. "Comparison with European quality indicators" and "Comparisons between high and low antibiotic prescribers"
**Discussion**				
Key results	18	Summarise key results with reference to study objectives	10-11	e.g. "We prospectively investigated patterns of antibiotic prescriptions over a two-and-a-half-year period in

For peer review only - http://bmjopen.bmj.com/site/about/guidelines.xhtml
For peer review only

				village clinics in rural Shandong province in order to assess evidence of irrational antibiotic use and variability in prescribing practices at the level of the individual prescriber.”
Limitations	19	Discuss limitations of the study, taking into account sources of potential bias or imprecision. Discuss both direction and magnitude of any potential bias	12-13	e.g. “A common limitation of studies using prescription data is the inability to verify diagnoses. To restrict the impact of this limitation we restricted the individual-level analyses to AURI diagnoses, but we cannot guarantee that misclassifications did not occur, particularly given there is currently no incentive for doctors to correctly classify diagnoses.” and entire section on methodological considerations
Interpretation	20	Give a cautious overall interpretation of results considering objectives, limitations, multiplicity of analyses, results from similar studies, and other relevant evidence	13	e.g. “Together our results provide evidence that even in a small homogenous setting in rural China, variations in individual prescriber practices are significant, and they need to be accounted for in the development of targets and interventions to improve antibiotic use.”
Generalisability	21	Discuss the generalisability (external validity) of the study results	13	e.g. “We suspect this finding is likely to be generalizable to other areas in rural Shandong province as

For peer review only

				a minimum, but also to other areas in eastern rural China, where the healthcare system is very similar in terms of structure, regulations and staffing.”
**Other information**				
Funding	22	Give the source of funding and the role of the funders for the present study and, if applicable, for the original study on which the present article is based	13	“This work was supported by the Swedish Research Council (grant number D0879801) and National Natural Science Foundation of China (grant number 81361138021).”

*Give information separately for cases and controls in case-control studies and, if applicable, for exposed and unexposed groups in cohort and cross-sectional studies.

Note: An Explanation and Elaboration article discusses each checklist item and gives methodological background and published examples of transparent reporting. The STROBE checklist is best used in conjunction with this article (freely available on the Web sites of PLoS Medicine at http://www.plosmedicine.org/, Annals of Internal Medicine at http://www.annals.org/, and Epidemiology at http://www.epidem.com/). Information on the STROBE Initiative is available at www.strobe-statement.org.

For peer review only

For peer review only - http://bmjopen.bmj.com/site/about/guidelines.xhtml
